# PRRC2A-mediated m^6^A modification on NCOA4 promotes thymic epithelial tumor progression through PKM2-induced glycolysis

**DOI:** 10.1038/s41419-026-08883-y

**Published:** 2026-07-08

**Authors:** Tao Wang, Lang Xia, Lei zhang, Yuntao Feng, Haoran E, Jueming Yu, Hongbo Huang, Junqi Wu, Chang Chen, Yue Zhao, Deping Zhao

**Affiliations:** 1 Department of Thoracic Surgery, Shanghai Pulmonary Hospital, School of Medicine, Tongji University, Shanghai, China; 2 Department of Thoracic Surgery; The Affiliated Suzhou Hospital of Nanjing Medical University, Suzhou Municipal Hospital Gusu School, Nanjing Medical University, Jiangsu, China; 3 Department of Thoracic Surgery, Ningbo No.2 Hospital, Ningbo, Zhejiang China

**Keywords:** Oncogenes, Tumour biomarkers, Epigenetics

## Abstract

Thymic epithelial tumors (TETs) are heterogeneous neoplasms. While most early-stage cases are curable after resection, advanced or aggressive subtypes have limited treatment options and poor outcomes. The molecular mechanisms underlying their metabolic reprogramming remain poorly understood. Proline-rich Coiled-coil 2a (PRRC2A), a N6-methyladenosine (m^6^A) reader protein, is significantly upregulated in TET tissues and is associated with poor clinical outcomes. Multi-omics analyses and molecular experiments revealed that PRRC2A binds to m^6^A-modified sites within the NCOA4 mRNA, thereby stabilizing NCOA4 transcripts and enhancing its protein expression. Elevated NCOA4 interacts with PKM2, a rate-limiting glycolytic enzyme, resulting in increased glycolysis and promoting TETs progression. In vivo experiments showed that inhibition of PRRC2A suppressed tumor growth and metastasis in TETs, while combined treatment with the PKM2 inhibitor C599 and PRRC2A knockdown produced enhanced antitumor effects. These findings provide novel insights into the glycolytic metabolic regulation of TETs and suggest that the PRRC2A-NCOA4-PKM2 axis represents a promising therapeutic target.

## Introduction

Thymic epithelial tumors (TETs), including thymoma and thymic carcinoma, are rare malignancies with limited therapeutic options for advanced disease [1, 2]. According to the current National Comprehensive Cancer Network (NCCN) clinical guidelines, the recommended chemotherapy regimen for TET (including both thymomas and thymic carcinomas) is a platinum-based combination therapy [3–6]. However, the overall objective response rate (ORR) is only 50–70%, and the complete response (CR) rate is less than 10% [7, 8]. Complete surgical resection remains the primary treatment modality [2]; nevertheless, for unresectable or metastatic cases, platinum-based chemotherapy represents the standard regimen but provides only modest clinical benefit [9, 10]. Therefore, further elucidation of the molecular mechanisms underlying TET initiation and progression is essential for identifying more effective therapeutic targets.

Epigenetic dysregulation has emerged as a critical driver of tumorigenesis across multiple cancer types. Among RNA modifications, N6-methyladenosine (m^6^A) is the most abundant internal modification of eukaryotic mRNA and plays a pivotal role in post-transcriptional gene regulation, including RNA stability, splicing, nuclear export, and translation [11–13]. The biological effects of m^6^A modification are mediated through a coordinated system of methyltransferases (“writers”), demethylases (“erasers”), and RNA-binding proteins (“readers”) [14–16]. Dysregulation of m^6^A signaling has been implicated in tumor initiation and progression, influencing processes such as proliferation, metastasis, and metabolic adaptation [11, 12, 17]. In TETs, recent studies have begun to implicate dysregulated m^6^A modification in tumor progression; however, the functional roles of individual m^6^A readers in this disease context remain largely unexplored.

Proline-rich coiled-coil 2A (PRRC2A) is a recently identified m^6^A reader protein that was initially reported to play an essential role in spermatogenesis. Subsequent studies have extended its functional relevance to cancer biology, demonstrating that PRRC2A promotes tumor progression in digestive system malignancies through activation of oncogenic signaling pathways [18–21]. Despite these emerging insights, whether PRRC2A contributes to TET pathogenesis and the molecular mechanisms by which PRRC2A-mediated m^6^A recognition regulates tumor-associated pathways in TETs remain undefined.

Nuclear receptor coactivator 4 (NCOA4) was initially characterized as a mediator of ferritinophagy and iron homeostasis [22–24]. Beyond its canonical role in iron metabolism, emerging evidence indicates that NCOA4 acts as a transcriptional coactivator and is critically involved in the regulation of gene expression in cancer cells. By cooperating with specific transcription factors, NCOA4 enhances the transcription of target genes, thereby orchestrating various cellular programs [25]. Notably, dysregulation of NCOA4 has been increasingly associated with the pathogenesis of various cancers. Emerging evidence positions NCOA4 as a critical regulator of metabolic reprogramming that drives tumor growth, particularly the shift toward aerobic glycolysis - known as the Warburg effect [26, 27]. This reprogramming allows cancer cells to preferentially generate energy and biosynthetic precursors rapidly, even under oxygen-sufficient conditions [28]. A central player in this glycolytic surge is the pyruvate kinase M2 isoform (PKM2), which is frequently upregulated in tumors [29]. As a key rate-limiting enzyme, PKM2 catalyzes the final step of glycolysis and diverts metabolic flux toward anabolic pathways required for cell proliferation [30]. Although recent studies suggest that NCOA4, through its core function as a transcriptional coactivator, may regulate the expression or activity of PKM2 either directly or indirectly, the precise mechanisms by which NCOA4-mediated facilitation of PKM2 function sustains the glycolytic phenotype, drives tumor bioenergetics, promotes biomass accumulation, and ultimately contributes to the progression and aggressiveness of TETs have not been elucidated.

In our study, we demonstrate that m^6^A modification and PRRC2A expression are both increased in thymic epithelial tumors, and that elevated PRRC2A expression is associated with more advanced clinicopathological features in TETs. Functional and mechanical studies revealed PRRC2A as a key m^6^A reader that stabilizes NCOA4 mRNA, which subsequently interacts with PKM2 to drive glycolytic reprogramming. Knockdown of PRRC2A or pharmacological inhibition of PKM2 markedly reduces TET growth and metastasis. These findings provide new insight into the metabolic regulation of TET and suggest that the PRRC2A-NCOA4-PKM2 axis could be a potential therapeutic target.

## Materials and methods

### Human specimens, mice, and study approval

This study comprised 12 TET tissues with their corresponding adjacent normal tissues, together with 58 additional thymic epithelial tumor specimens collected from Shanghai Pulmonary Hospital. The clinical characteristics of the 54 patients included in the clinicopathological analysis are summarized in Table S1. All participants signed written informed consent prior to sample collection. Male BALB/c nude mice aged 4–6 weeks were obtained from the Animal Center of Shanghai Pulmonary Hospital. Ethical approval for this research was granted by the Institutional Review Board of Shanghai Pulmonary Hospital (No. K24-567).

### Cell culture

The Ty-82 cell line, originally established from a tumor diagnosed as thymic carcinoma at the time of derivation, was kindly provided by Prof. Ze-Hong Miao from the Shanghai Institute of Materia Medica, Chinese Academy of Sciences. Subsequent molecular characterization showed that Ty-82 harbors a BRD4-NUT fusion and is thus consistent with NUT carcinoma of the thorax, a rare molecularly defined subtype within thymic epithelial tumors. The IU-TAB-1 cell line, derived from type AB thymoma, was sourced from Applied Biological Materials Inc. (Abm, Canada). The ThyL-6 cell line, derived from an undifferentiated human thymic carcinoma, was obtained from the International Patent Organism Depositary (IPOD, NITE, Japan), with assistance from the Research Promotion Division of the University of Fukui. HEK 293T cells (human embryonic kidney cells expressing the SV40 large T antigen; obtained from the Cell Bank of the Chinese Academy of Sciences, Shanghai, China) were used exclusively for lentiviral packaging. Cells were maintained in DMEM supplemented with 10% FBS and 1% penicillin-streptomycin at 37 °C in 5% CO₂. Ty-82 and ThyL-6 cells were cultured in RPMI 1640 medium (Procell, China), IU-TAB-1 cells and HEK 293T were grown in DMEM (Procell), both supplemented with 10% fetal bovine serum (FBS) and 1% penicillin-streptomycin (Hyclone, Cytiva, USA). All cells were maintained at 37 °C in a 5% CO_2_ atmosphere and periodically tested for mycoplasma.

### Transfection and construction of stable overexpression and knockdown TET cell lines

Transfection procedures were performed using Lipofectamine 3000 (Invitrogen, USA), following the manufacturer’s guidelines. Cells were collected 48 h post-transfection for downstream analyses. PRRC2A knockdown and overexpression lentiviruses were purchased from OBiO Technology (Shanghai, China). First, TET cells were seeded in a six-well plate. Cells were seeded in six-well plates and transduced upon reaching 70% confluence. After 24 h of infection, the media were replaced with fresh medium containing 10 µg/mL puromycin to select stably transduced cells. Complete medium was added with 10 µg/mL puromycin for continuous delivery. The shRNA sequences are listed in Table S2.

### Western blot

Tissues or cultured cells in 10 cm dishes were lysed using RIPA buffer (Beyotime, China) supplemented with protease and phosphatase inhibitors. Protein concentration was determined with a BCA assay (Beyotime, China), and equal amounts were resolved by 10% SDS-PAGE and transferred onto PVDF membranes (Merck, Germany). Membranes were blocked for 1 h at room temperature with 5% skim milk in TBST. Primary antibodies were incubated overnight at 4 °C, followed by HRP-conjugated secondary antibodies (Servicebio, China) for 1 h at room temperature. Signal detection was achieved using an ECL kit (Enlighten biotech, China). Primary antibodies were listed in Table S3.

### Real-time reverse transcription-quantitative polymerase chain reaction (RT-qPCR)

Total RNA from tissues or TET cell lines was isolated with TRIzol reagent (Thermo Scientific, USA), and concentrations were measured using a Nanodrop 1000. cDNA synthesis was performed using PrimeScript RT reagent Kit (Takara, Japan). RT-qPCR was carried out on an ABI PRISM 7300HT Sequence Detection System using TB Green Premix Ex Taq II FAST qPCR Kit (Takara, Japan), and gene expression was quantified using the 2^−ΔΔCt^ method. The primer sequences used for RT-qPCR are listed in Table S4.

### Immunohistochemistry (IHC)

Paraffin-embedded tissues were sliced into thin sections and deparaffinized in dimethylbenzene and alcohol at different concentrations. The sections were then repaired with sodium citrate, blocked with goat serum, and incubated with Primary antibodies at 4 °C overnight. The following day, the sections were incubated with horseradish peroxidase-conjugated secondary antibodies (Servicebio, China) and incubated with DAB solution (Servicebio, China). Hematoxylin solution (Servicebio, China) was used to stain the nuclei. Primary antibodies were listed in Table S3. Immunohistochemical staining was independently assessed by two experienced pathologists with expertise in thymic tumor histopathology, who were blinded to the clinical information. Discrepancies in cell identification or scoring were resolved by consensus review. Representative images were selected from areas with clearly discernible epithelial tumor components to minimize potential interference from lymphocyte-rich regions.

Protein expression was evaluated using a semi-quantitative scoring system based on staining intensity and the proportion of positively stained tumor cells. Staining intensity was graded as 0 (negative), 1 (weak), 2 (moderate), or 3 (strong). The proportion of positive tumor cells was categorized as 0 (0–5%), 1 (6–25%), 2 (26–50%), 3 (51–75%), or 4 (76–100%). A composite score ranging from 0 to 12 was calculated for each case by multiplying the intensity score by the percentage category score. Patients were stratified into low and high-expression groups using the median composite score within the cohort as the cutoff. Cases with scores equal to the median value were assigned to the low-expression group. This cutoff was applied uniformly across all subsequent correlation and survival analyses.

### Cell counting kit-8 (CCK-8) assay

TET cells were seeded into 96-well plates at a density of 5 × 10^3^ cells per well for cultivation. Subsequently, the culture medium was replaced with a medium containing 10% CCK-8 reagent (Elabscience, Wuhan, China) at various time points (12, 24, 48, and 72 h), and incubated for 1 h. The absorbance of each sample was then quantified at 450 nm.

### Colony formation

2 × 10^3^ TET cells were seeded in a 6-well plate and cultured for 14 d. The colonies were fixed with 4% neutral paraformaldehyde and stained with 0.1% crystal violet for 10 min at room temperature. After washing to remove floating color, the colonies were counted using ImageJ software.

### Cell migration and invasion assay

For the cell migration assay, 1 × 10^5^ TET cells were suspended in 200 µL of FBS-free medium and placed in the upper chamber of the transwell filter. The lower chamber of the filter was filled with 600 µL of 10% FBS medium. After 48 h of culture, cells migrating through the filter were fixed with 4% paraformaldehyde and stained with a crystal violet solution (Solarbio, China). For the cell invasion assay, the front side of the filter was pre-coated with Matrigel matrix. Subsequently, 1 × 10^5^ cells were placed in the upper chamber, and cells invading the filter were fixed and stained. The average number of cells in five fields was calculated using ImageJ software. The results are representative of three independent experiments.

### Xenograft model

For in vivo tumor growth, four-week-old BALB/c nude mice were used. TET cells (1 × 10^7^ cells in 100 µL PBS) were injected subcutaneously into the right flank. Tumors were collected 2 months post-injection, and the tumor length, width, and weight were measured. The tumor volume (mm^3^) was calculated as follows: (longest diameter) × (shortest diameter)^2^ × (π/6).

### Lung metastasis model

In the lung metastasis model, mice were injected intravenously with 150 µL of cell suspension containing 1 × 10^6^ cells via the tail vein. After 2 months, lungs were harvested, and metastatic nodules were counted.

### N6-methyladenosine-modified RIP

MeRIP-seq was executed in the following manner: Initially, we isolated total RNA utilizing the Trizol reagent (Takara, Japan), which was followed by an mRNA purification process implemented through the GenEluteTM mRNA Miniprep Kit (Sigma, Louis, MO). Following this, we deployed RNA fragmentation reagents (NEB, UK) to fragment the RNA in a random manner. During the immunoprecipitation procedure, a specific anti-m^6^A antibody (Synaptic Systems, 202003) was previously combined with Protein G magnetic beads in a reaction buffer. The fragmented mRNA was then exposed to a 4 °C atmosphere for an hour-long incubation with the mixture. After a washing process using low salt and high salt reaction buffers, Buffer RLT (Qiagen, Hilden, Germany) was used to extract the m^6^A-antibody-bound RNA from the Dynabeads. A further incubation was conducted with Dynabeads MyOne Silane (Life Technologies, West Palm Beach, FL). The precipitation was completed with 100% ethanol, a 70% ethanol wash, and a resuspension of the RNA and Dynabeads mixture in nuclease-free water. The supernatant, containing the m^6^A-bound RNA, was carefully collected following bead separation with a magnetic field.

### RNA-seq

RNA-sequencing experiments were performed following standard protocols. Total RNA was extracted, quality-checked, and libraries were prepared using the NEBNext® Ultra™ II RNA Library Prep Kit for Illumina® (New England Biolabs, USA). Paired-end libraries (2 × 150 bp) were sequenced on an Illumina NovaSeq 6000 platform (Illumina, USA) by OE Biotech Co., Ltd. (Shanghai, China). Sequencing was conducted on a high-throughput platform, and raw reads were aligned to the reference genome. Differential gene expression was analyzed using DESeq2.

### Immunofluorescence (IF) staining

For immunofluorescence, TET cells were seeded on glass coverslips in 24-well plates. The next day, cells were fixed with 4% paraformaldehyde, permeabilized using 0.5% Triton X-100, and blocked in 5% goat serum. After washing, primary antibodies were incubated overnight at 4, followed by fluorescence-labeled secondary antibodies at room temperature. Nuclei were stained with DAPI, and images were obtained using an Olympus inverted fluorescent microscope. Primary antibodies were listed in Table S3.

### RIP-qPCR and RIP-seq

For RIP-qPCR and RIP-seq, 2 × 10^7^ TET cells were washed with cold PBS and lysed in RIP buffer with RNase inhibitors. Lysates were cleared by centrifugation, and a portion was saved as input. The remainder was incubated with Protein A Dynabeads conjugated to anti-PRRC2A or IgG control antibodies. After incubation and washing, RNA-protein complexes were eluted, treated with proteinase K, and RNA was extracted using TRIzol. Purified RNAs were analyzed via qPCR or sequenced.

### RNA pull-down assay

RNA pull-down assays were conducted using the Pierce Magnetic RNA-Protein Pull-Down Kit (Thermo Fisher). Biotin-labeled NCOA4 RNA probes were incubated with cell lysates and streptavidin magnetic beads. After washing and elution, proteins were analyzed by Western blot using anti-PRRC2A antibody.

### Dual-luciferase reporter assays

Dual-luciferase reporter assays followed Promega’s Dual-Glo protocol. A 2-kb PRRC2A promoter fragment containing or lacking ATF1-binding sites was cloned into pGL3-basic vectors and co-transfected with phRL-TK into Ty-82 cells. Mutations at ATF1-binding regions were introduced by site-directed mutagenesis. After 36 h, firefly and Renilla luciferase activities were measured. Ratios of firefly to Renilla signals were normalized to control values. To assess PRRC2A regulation of NCOA4 via the 3′UTR, wild-type or mutant NCOA4-UTR plasmids were co-transfected with PRRC2A siRNA or overexpression plasmids. Luciferase activity was measured after 36 h and analyzed as above. Normalization was performed using Renilla luciferase.

### mRNA stability assay

For mRNA stability, actinomycin D (5 µg/mL) was added to transfected TET cells at time points of 0, 2, 4, 6, and 8 h. Total RNA was extracted, reverse-transcribed, and subjected to RT-qPCR to quantify target mRNA degradation.

### m^6^A dot blot

The GenEluteTM mRNA Miniprep Kit (Sigma, MO) was utilized on pre-extracted total RNA to isolate polyadenylated mRNA. Following this, the poly(A) + RNA samples were positioned onto a Hybond-N + membrane (GE Healthcare, UK) and subsequently crosslinked with UV. The membrane was then subjected to blocking with 5% skim milk for one hour, followed by overnight incubation at 4 °C with m^6^A antibody (Synaptic Systems, 202003). The membrane then underwent probing with anti-mouse IgG, and signal visualization was accomplished with the ECL detection system. For comparison, an equal volume of poly(A)^+^ RNA samples was applied to the membrane and stained with 0.02% methylene blue (MB) in 0.3 M sodium acetate (pH = 5.2). The ImageJ software was leveraged to quantify the relative signal intensity of each dot. The results pertaining to m^6^A levels were demonstrated as the relative m^6^A dot blot intensity standardized to the methylene blue staining density.

### Extracellular acidification rate (ECAR) measurement

ECAR values were measured using a Seahorse XF24 Extracellular Flux analyzer (Seahorse Bioscience, USA) to quantify glycolysis in TET cells. Before the experiment, the culture medium was replaced with fresh KRPH medium. Glucose, oligomycin, and 2-deoxyglucose (2-DG) were then added to ports A-C, respectively, and the program was started to measure. Each procedure consisted of mixing, spacing, and measurement, and the result of each measurement cycle was the ECAR value at a specific point.

### Measurements of glycolysis-related indices

For the measurement of fructose-1,6-diphosphate (FDP) content, LDH activity, lactate level, and acetyl-CoA content, cells were lysed using ultrasound and centrifuged at 12,000 × *g* for 10 min. The supernatant was collected, supplemented with working solutions, and centrifuged repeatedly. The supernatant was collected, and the absorption value was measured using a spectrophotometer. For the measurement of glucose uptake, the culture medium was collected before and after the cells were cultured for 24 h to assess the level of glucose uptake. FDP content, 540 nm (Solarbio, China); LDH activity, 450 nm (Solarbio, China); glucose uptake, 505 nm (Solarbio, China); lactate level, 570 nm (Solarbio, China); and acetyl-CoA content, 340 nm (Abbkine, China).

### EDU

Cell proliferation was detected using a 5-ethynyl-2′-deoxyuridine (EdU) kit (Ribobio, China). EdU solution (10 µM) was added into cells in the logarithmic phase of growth and incubated at 37 °C, 5% CO_2_. After 2 h, cells were fixed with 4% neutral paraformaldehyde and permeabilized with 0.5% Triton X-100. Next, Apollo 643 was added to the cells and incubated at 25 °C for 30 min in the dark. After repeated washes, the nuclei were stained with Hoechst 33342. The EdU-positive cells fluoresced red, and the proportion of EdU-positive cells among all cells was analyzed using ImageJ. The results are representative of three independent experiments.

### Co-immunoprecipitation (Co-IP)

A PierceTM co-IP kit (Thermo, USA) was used to identify the interaction between NCOA4 and PKM2. Briefly, primary antibodies and magnetic beads were incubated at 25 °C for 15 min with constant rotation for conjugation. Cells were lysed with RIPA solution and centrifuged at 13,000 × *g* for 15 min to acquire the proteins. The total protein solution was incubated with conjugated antibodies at 25 °C for 1 h. After elution, the supernatant containing the target antigen was collected and analyzed using Western blot and mass spectrometry.

### Mass spectrometry

Proteins that directly interacted with NCOA4 were obtained using the co-IP assay. Proteins were isolated using SDS-PAGE. Follow-up treatment was performed when protein bands were visible. The gel was then decolorized using a decolorizing solution, reduced with dithiothreitol, and alkylated with iodoacetamide. The protein was digested into polypeptides with trypsin at 37 °C overnight, then the peptides were extracted and desalted. Peptides were identified using an EASY-nLC 1000 (Thermo, USA) coupled with Orbitrap Fusion Lumos (Thermo, USA). Peptide information was matched against the SwissProt database using Proteome Discovery 2.4 software (Thermo, USA).

### Statistical analysis

All experiments were conducted in triplicate unless otherwise noted. Data are presented as mean ± standard deviation (SD). Comparisons between two groups were performed using Student’s *t*-test for normally distributed data or the Mann–Whitney *U*-test for nonparametric data. Comparisons among multiple groups were performed using one-way ANOVA followed by Tukey’s post hoc test for normally distributed data or the Kruskal–Wallis test for nonparametric data. *P*-value < 0.05 was considered statistically significant (*P* < 0.05; *P* < 0.01).

## Results

### Single-cell analysis identifies PRRC2A as a key regulator in TET and links its overexpression to poor prognosis

To systematically investigate the epithelial compartment and intratumoral heterogeneity of TETs, we integrated and analyzed publicly available single-cell RNA sequencing (scRNA-seq) datasets comprising tumor samples from 6 TET patients and 1 normal thymus sample (GSE147520, HRA002334). These analyses were further validated using our own patient samples.

**Fig. 1:**
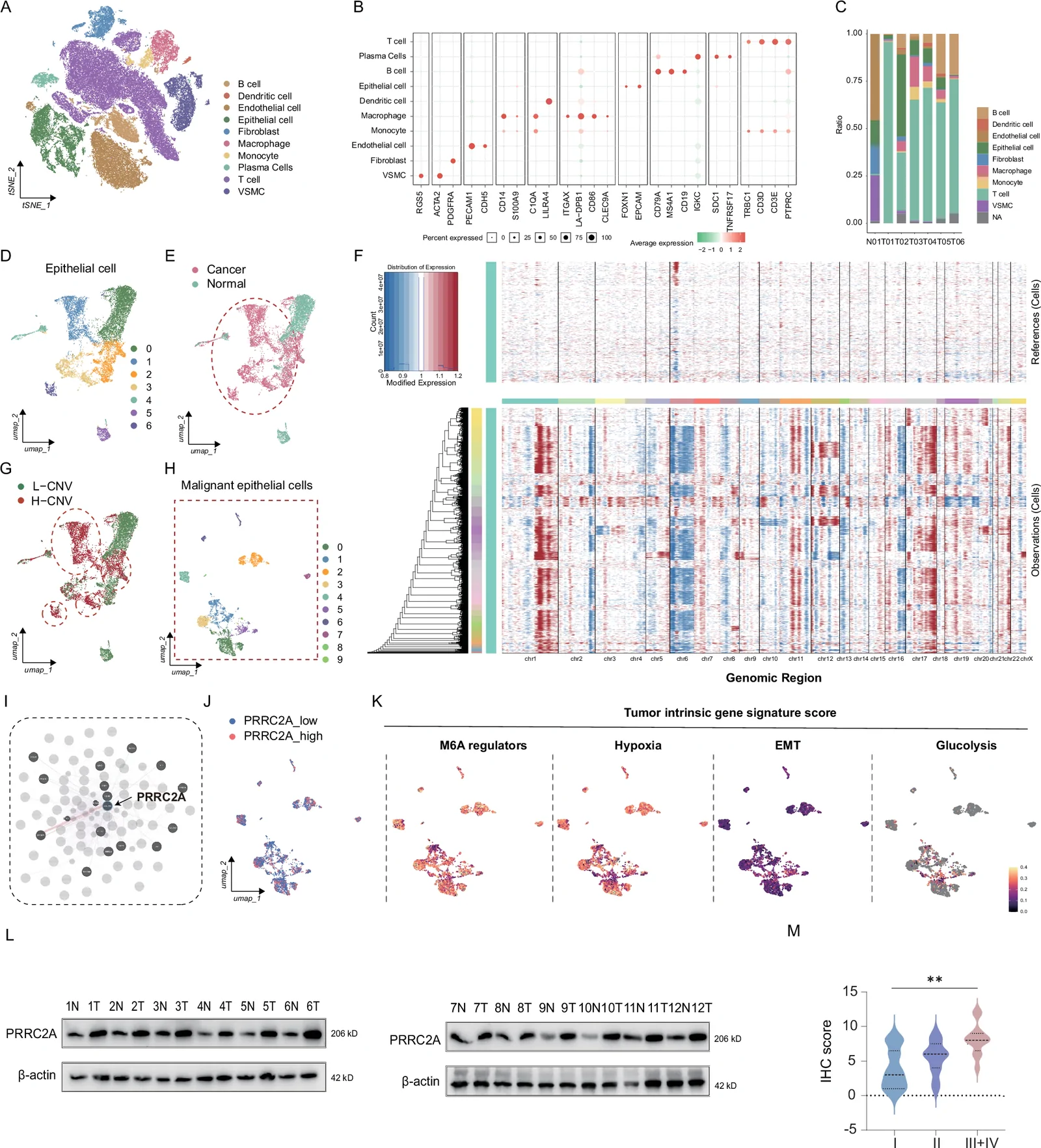
Single-cell analysis identifies PRRC2A as a key regulator in TET and links its overexpression to poor prognosis. **A** UMAP visualization of single-cell RNA-seq data from 6 TET samples and 1 normal thymus sample, revealing major cell populations, including T cells, B cells, dendritic cells, epithelial cells, fibroblasts, endothelial cells, macrophages, monocytes, plasma cells, and vascular smooth muscle cells (VSMCs). **B** Dot plot showing the expression patterns of canonical marker genes across different cell populations for cell type annotation and identification. **C** Stacked bar plot illustrating the proportions of different cell types, highlighting changes in the immune microenvironment between normal thymus and TET samples. **D** Re-clustering of epithelial cells extracted from the single-cell dataset, demonstrating marked intratumoral heterogeneity. **E** Classification of epithelial cells based on tissue origin, distinguishing tumor-derived epithelial cells from normal epithelial cells. **F** Copy number variation (CNV) analysis of epithelial cells using the InferCNV algorithm. The upper heatmap shows stable CNV patterns in normal epithelial cells, while the lower heatmap reveals genomic instability in tumor-derived epithelial cells. **G** Tumor-derived epithelial cells were further subdivided into high CNV (H-CNV, red) and low CNV (L-CNV, green) populations according to CNV scores. **H** Integration of tissue origin and CNV status defined malignant epithelial cells, which were subsequently re-clustered for downstream analyses. **I** Differential expression and network analysis identified PRRC2A as a central hub gene in malignant epithelial cells. **J** UMAP plot showing the spatial distribution of PRRC2A expression, revealing PRRC2A^high^ and PRRC2A^low^ tumor epithelial subpopulations. **K** Enrichment analysis of PRRC2A^high^ cells demonstrating strong associations with m^6^A regulatory pathways, hypoxia, epithelial–mesenchymal transition (EMT), and glycolytic signatures. **L** Western blot analysis of PRRC2A protein levels in 12 representative pairs of TET and normal tissues, with β-actin as a control. **M** Association between PRRC2A expression and overall pathological stage (*n* = 54). Statistical significance was analyzed using the Kruskal–Wallis test followed by the Mann–Whitney *U*-test for pairwise comparisons. **P < 0.01.

We first performed global clustering of all cells from the integrated datasets. UMAP analysis revealed ten major cell populations, including B cells, dendritic cells, endothelial cells, epithelial cells, fibroblasts, macrophages, monocytes, plasma cells, T cells, and vascular smooth muscle cells (VSMCs) (Fig. 1A). To accurately define each population, we visualized the expression of canonical marker genes across these clusters using a dot plot, enabling precise cell type annotation (Fig. 1B). A stacked bar plot further highlighted the differences in immune microenvironment composition between normal thymus and TET samples, revealing a marked increase in malignant epithelial cells and a shift in immune cell proportions in tumors (Fig. 1C). From these datasets, epithelial cells were then extracted and re-clustered, revealing marked heterogeneity within the epithelial compartment (Fig. 1D). Based on tissue origin, epithelial cells were classified into normal epithelial cells and tumor-derived epithelial cells, clearly distinguishing benign from malignant tissue sources (Fig. 1E).

To further delineate genomic features, we performed copy number variation (CNV) analysis using the InferCNV algorithm. This revealed extensive chromosomal instability among tumor-derived epithelial cells compared with normal epithelial cells, a hallmark of malignant transformation (Fig. 1F). According to CNV scores, tumor-derived epithelial cells were subdivided into high CNV (H-CNV) and low CNV (L-CNV) groups, providing a refined classification of malignant potential (Fig. 1G). By integrating tissue origin and CNV status, we defined a population of malignant epithelial cells, which were subsequently re-clustered to enable downstream transcriptional and functional analyses (Fig. 1H). To identify potential key regulators driving malignant transformation, we combined differential expression analysis with gene co-expression network analysis (WGCNA). Among the top-ranked hub genes, PRRC2A consistently exhibited strong upregulation and the highest network centrality, indicating a potential pivotal role in coordinating tumor-associated transcriptional programs (Fig. 1I). Given that PRRC2A encodes an m6A reader protein previously implicated in cancer progression, we focused subsequent analyses on elucidating its function in TETs. Spatial mapping of PRRC2A expression further revealed distinct PRRC2A⁺ and PRRC2A⁻ subpopulations within the malignant epithelial compartment (Fig. 1J). Enrichment analysis of PRRC2A⁺ cells showed significant activation of key oncogenic pathways, including m^6^A RNA modification regulation, hypoxia response, epithelial–mesenchymal transition (EMT), and glycolysis programs, indicating that PRRC2A⁺ cells represent a transcriptionally distinct and potentially more aggressive subset of malignant epithelial cells in TETs (Fig. 1K).

To validate the expression pattern of PRRC2A in TET, we analyzed 12 paired tumor and adjacent normal thymic tissues, as well as 42 additional thymic epithelial tumor samples collected from Shanghai Pulmonary Hospital. Western blot analysis demonstrated significantly elevated PRRC2A protein levels in tumor tissues compared with adjacent normal tissues (Fig. 1L and Fig. S1A). Consistently, immunohistochemistry (IHC) showed markedly higher PRRC2A staining scores in tumor samples, confirming its overexpression in TET (Fig. S1B). We then assessed the clinical significance of PRRC2A expression. High PRRC2A levels were significantly associated with advanced TNM stage (Fig. 1M).

Collectively, these findings indicate that PRRC2A is a critical gene enriched in high-risk tumor epithelial subpopulations and is strongly associated with aggressive clinicopathological characteristics in TETs, suggesting its potential role as both a biomarker and a driver of tumor progression.

### PRRC2A enhances the proliferative and metastatic capacity of TET cells in vitro and in vivo

**Fig. 2:**
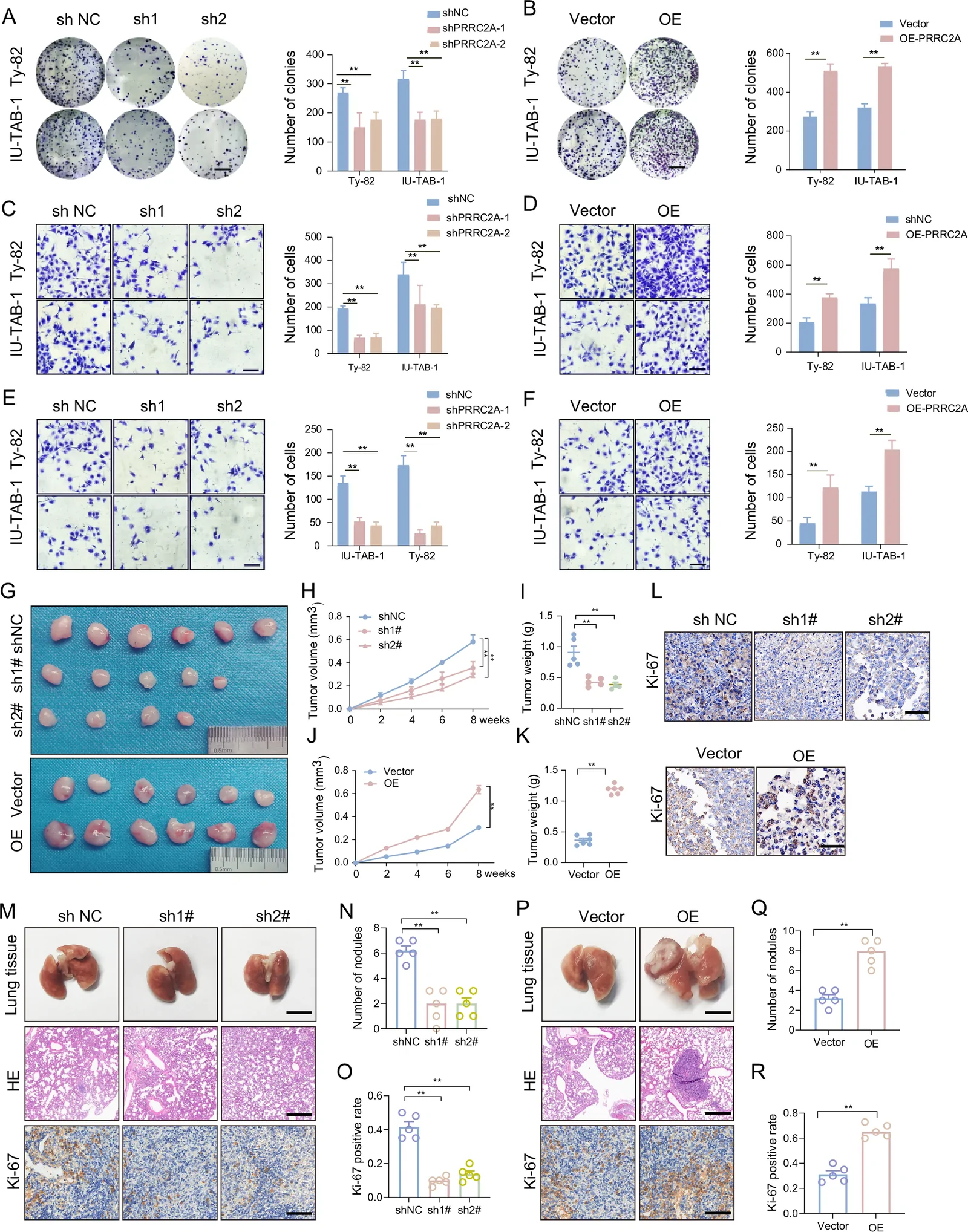
PRRC2A enhances the proliferative and metastatic capacity of TET cells in vitro and in vivo. Colony formation assays revealed the proliferation of TET cells with knockdown (**A**) or overexpression (**B**) of PRRC2A. Representative graphs are presented (Scale bars: 5 mm). The quantifications of cell clone spheres are presented. *n* = 3 in each group. Statistical significance was analyzed using Student’s *t*-test. Transwell assays were employed to evaluate the migration ability of TET cells with knockdown (**C**) or overexpression (**D**) of PRRC2A. Representative micrographs are presented (Scale bars: 50 μm). The quantifications of cell clone spheres are presented. *n* = 3 in each group. Statistical significance was analyzed using Student’s *t*-test. Transwell assays were employed to evaluate the invasion ability of TET cells with knockdown (**E**) or overexpression (**F**) of PRRC2A. Representative micrographs are presented (Scale bars: 50 μm). The quantifications of cell clone spheres are presented. *n* = 3 in each group. Statistical significance was analyzed using Student’s *t*-test. **G** Subcutaneous xenograft tumors from BALB/c nude mice injected subcutaneously with PRRC2A knockdown or overexpression TET cells. The volume and weight of the Subcutaneous xenograft tumors from BALB/c nude mice injected subcutaneously with PRRC2A knockdown (**H**, **I**) or overexpression (**J**, **K**) Ty-82 cells. *n* = 6. Tumor growth curves were analyzed using two-way repeated-measures ANOVA, and endpoint tumor weights were analyzed using Student’s *t*-test. **L** Representative images of immunohistochemistry (Ki-67) to evaluate the proliferative ability of Subcutaneous xenograft tumors (Scale bar: 50 μm). **M**–**O** Representative images of surgically removed lung tissues (Scale bar: 5 mm) and immunohistochemical results of H&E staining (Scale bar: 200 μm) and Ki-67 staining (Scale bar: 100 μm) with PRRC2A knockdown Ty-82 cells. *n* = 5 in each group. Quantification of histological and immunohistochemical data was analyzed using Student’s *t*-test. **P**–**R** Representative images of surgically removed lung tissues (Scale bar: 5 mm) and immunohistochemical results of H&E staining (Scale bar: 200 μm) and Ki-67 staining (Scale bar: 100 μm) with PRRC2A overexpression Ty-82 cells. *n* = 5 in each group. Statistical analysis of the quantified data was performed using Student’s *t*-test. *P < 0.05, **P < 0.01.

To further explore the potential biological roles of PRRC2A in TET. Stable PRRC2A-knockdown and PRRC2A-overexpressing Ty-82, IU-TAB-1, and ThyL-6 cell lines were established using lentiviral transduction, respectively (Fig. S1C–E). Firstly, CCK-8 assays demonstrated that PRRC2A knockdown significantly reduced TET cells proliferation compared with controls, whereas PRRC2A overexpression markedly enhanced TET cells proliferation (Fig. S1F–H). This finding was further confirmed by colony formation assays, which revealed markedly fewer colonies in PRRC2A-knockdown TET cells and more colonies in PRRC2A-overexpressing TET cells (Fig. 2A, B; Fig. S1I–K). Next, to further investigate the role of PRRC2A in the metastasis of TET cells, transwell assays were performed. Knockdown of PRRC2A significantly reduced the migration and invasive capabilities of TET cells, whereas its overexpression markedly enhanced these phenotypes (Fig. 2C–F and Fig. S1L–O). These findings collectively demonstrate that PRRC2A enhances both proliferative and metastatic characteristics of TET cells in vitro.

To investigate the in vivo role of PRRC2A in TET cells, we utilized a subcutaneous xenograft model in Balb/c mice to assess the impact of PRRC2A on the proliferative capacity of Ty-82 cells. The results demonstrated that, compared to the control group, knockdown of PRRC2A in TET cells led to significantly reduced tumor volume and tumor weights in subcutaneous xenografts (Fig. 2G–I). In contrast, overexpression of PRRC2A in Ty-82 cells resulted in a marked increase in both tumor volume and tumor weight compared with the control group (Fig. 2J, K). Immunohistochemical analysis of tumor tissue for Ki-67 revealed that the Ki-67 positivity rate in subcutaneous tumors derived from PRRC2A-knockdown Ty-82 cells was significantly decreased compared to the control group. Conversely, tumors from PRRC2A-overexpressing Ty-82 cells showed a significant increase in Ki-67 positivity compared with the control group (Fig. 2L). Additionally, a tail vein injection lung metastasis model was employed to evaluate the effect of PRRC2A on the metastatic potential of Ty-82 cells. The results indicated that, relative to the control group, knockdown of PRRC2A in Ty-82 cells significantly reduced the number of metastatic nodules and the Ki-67 positivity rate within metastatic lesions (Fig. 2M–O). In contrast, overexpression of PRRC2A in Ty-82 cells led to a significant increase in the number of metastatic nodules and the Ki-67 positivity rate compared with the control group (Fig. 2P–R).

Together, these results indicate that PRRC2A functions as an oncogenic regulator that drives TET cells' proliferation and metastasis both in vitro and in vivo.

### Identification of the PRRC2A downstream target by multi-omics analysis

**Fig. 3:**
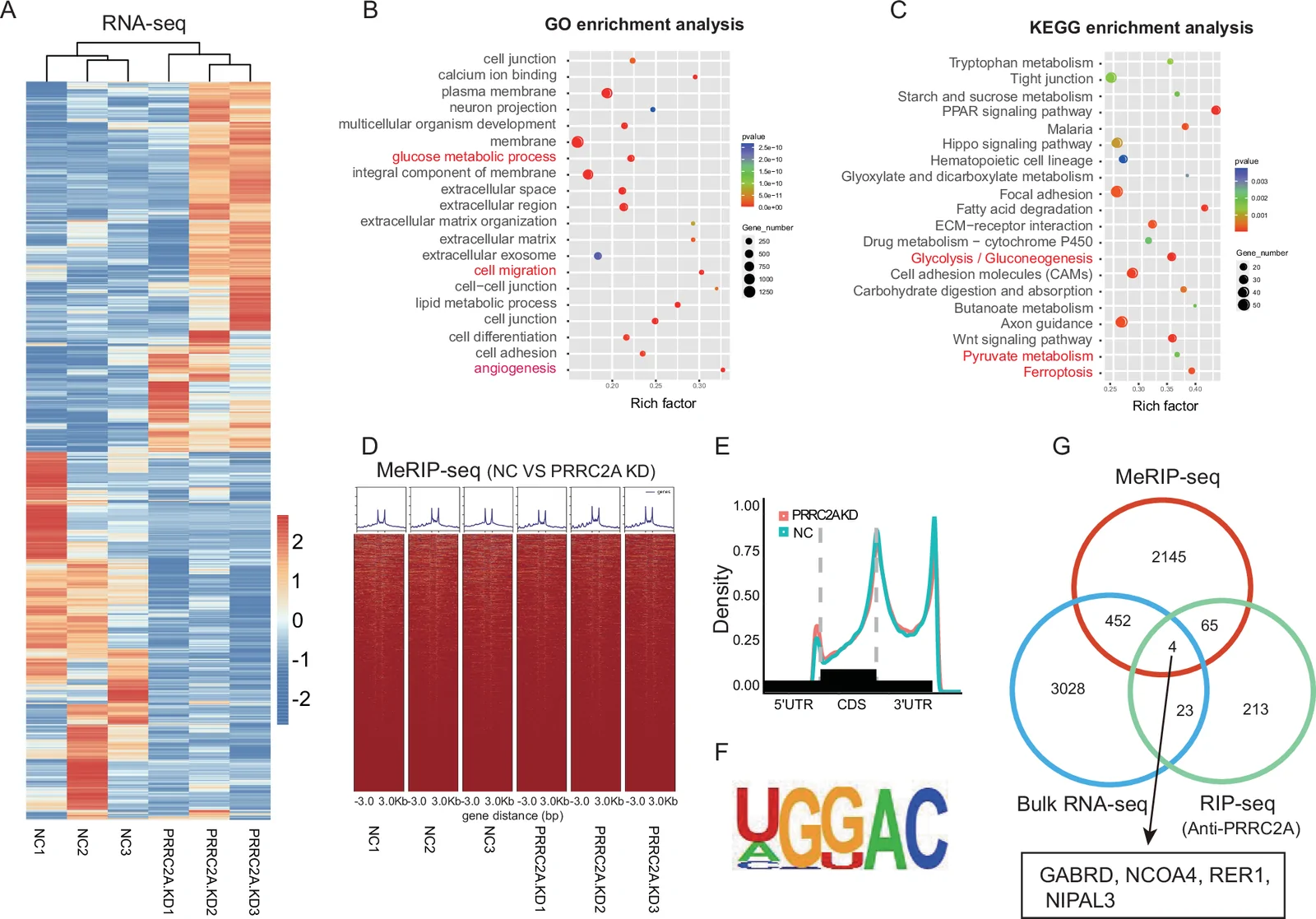
Identification of the PRRC2A downstream target by multi-omics analysis. **A** Heatmap displaying normalized expression values of genes identified by bulk RNA-seq analysis, comparing Ty-82 cells with PRRC2A knockdown to control cells. Enrichment Analysis illustrating significantly enriched biological processes (**B**) and signaling pathways (**C**) following PRRC2A knockdown. **D** MeRIP-seq analysis conducted in Ty-82 cells with stable PRRC2A knockdown and corresponding controls. **E** Distribution of m^6^A modification peak densities across distinct RNA regions in Ty-82 cells with stable PRRC2A knockdown versus controls. **F** Motif analysis identifying consensus sequences enriched for m^6^A modification in Ty-82 cells. **G** Venn diagram depicting the overlap of significantly altered genes among bulk RNA-seq, MeRIP-seq, and RIP-seq datasets.

In order to elucidate the molecular mechanisms through which PRRC2A promotes TET progression, we performed RNA-seq in PRRC2A-knockdown and control Ty-82 cells. Analysis of the RNA-seq data identified 2023 downregulated and 1720 upregulated genes (*q*-value < 0.05) in the PRRC2A-KD group compared with the control group (Fig. 3A). Gene Ontology (GO) analysis of these PRRC2A-regulated transcripts revealed significant enrichment in metabolic processes, particularly glucose metabolism, as well as pathways involved in cell migration and angiogenesis (Fig. 3B). Kyoto Encyclopedia of Genes and Genomes (KEGG) pathway analysis further demonstrated enrichment in glycolysis and pyruvate metabolism (Fig. 3C). These results suggest that PRRC2A plays an important role in the regulation of tumor metabolism and cancer progression.

Given the established role of PRRC2A as an m^6^A reader [19, 20], m6A RNA immunoprecipitation sequencing (MeRIP-seq) was performed to profile transcriptome-wide m6A modifications in both the PRRC2A-KD and control groups (Fig. 3D), Consistent with previous reports [31]. We observed a predominant enrichment of m6A peaks near the stop codon regions in both groups, and PRRC2A knockdown did not alter the global m6A modification levels (Fig. 3E). Motif analysis further revealed a conserved “GGAC” sequence within these enriched regions (Fig. 3F).

To identify transcripts directly bound by PRRC2A, we integrated RNA-seq, MeRIP-seq, and RIP-seq datasets. Considering that PRRC2A has been previously reported to play a critical role in regulating mRNA stability, we first intersected the differentially expressed genes identified by MeRIP-seq and RNA-seq to screen for transcripts whose expression changes were associated with altered m^6^A modifications. Next, we overlapped these candidates with the targets identified by anti-PRRC2A RIP-seq. Through this integrative approach, we defined a set of downstream mRNAs whose expression was potentially regulated by PRRC2A in an m6A-dependent manner. This analysis revealed 456 genes that were differentially expressed upon PRRC2A knockdown compared with the control group. Further integration with the RIP-seq dataset highlighted GABRD, NCOA4, RER1, and NIPAL3 as the most significantly enriched PRRC2A-bound targets (Fig. 3G).

### PRRC2A maintain NCOA4 mRNA stability in an m^6^A-dependent manner

**Fig. 4:**
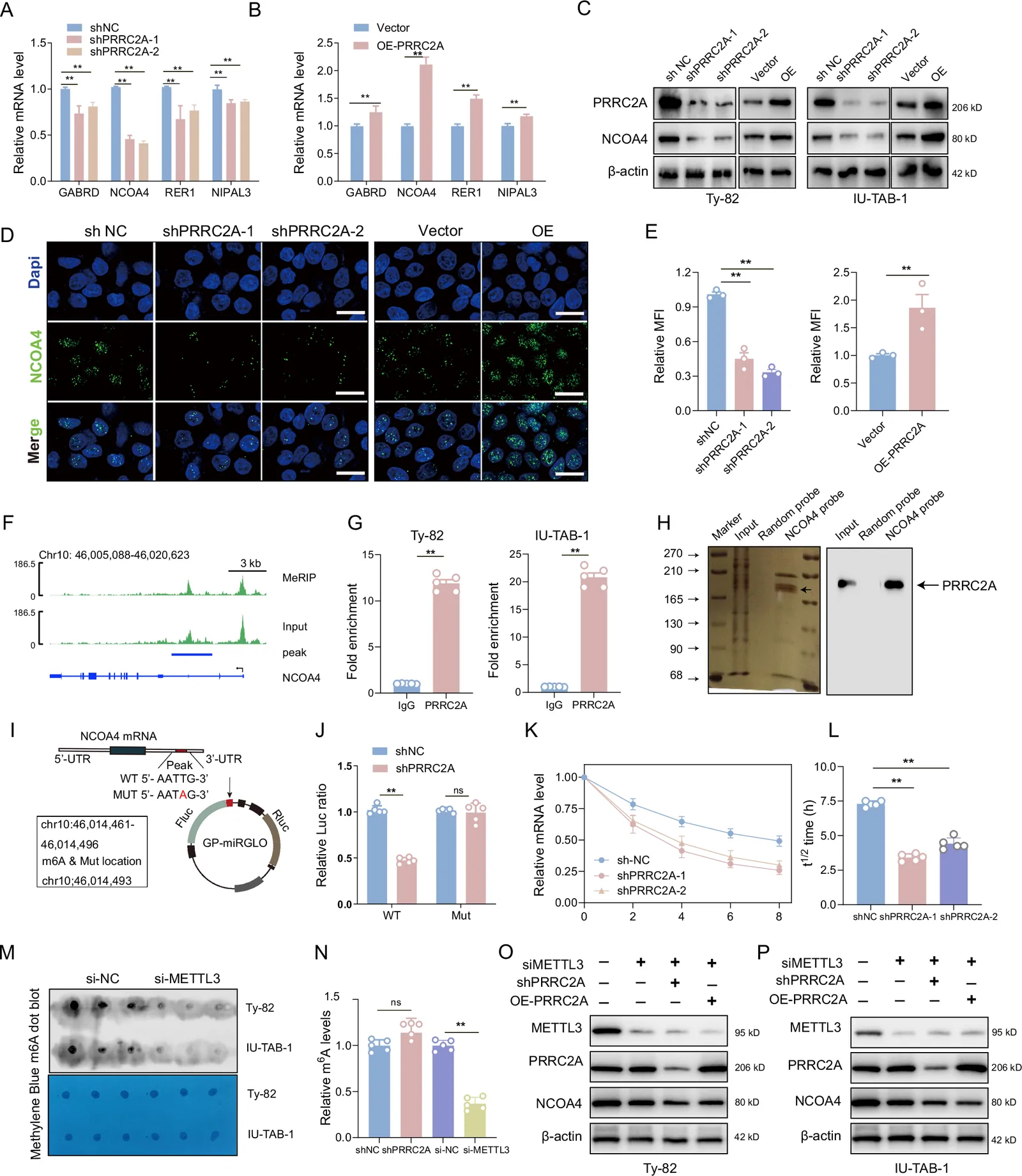
PRRC2A maintains NCOA4 mRNA stability in an m^6^A-dependent manner. Relative mRNA levels of four candidate genes, assessed by RT-qPCR in Ty-82 cells following PRRC2A knockdown (**A**) or overexpression (**B**), validating the regulatory relationship between PRRC2A and these targets. *n* = 3 in each group. Statistical analysis of the quantified data was performed using Student’s *t*-test. **C** Western blot analysis confirming changes in NCOA4 protein expression in TET cells upon PRRC2A knockdown or overexpression. **D**, **E** Immunofluorescence (IF) analysis validating alterations in NCOA4 protein levels (green fluorescence) in TET cells with PRRC2A knockdown or overexpression (Scale bar: 10 μm). *n* = 3 in each group. Statistical analysis of the quantified data was performed using Student’s *t*-test. **F** m^6^A binding sites on NCOA4 mRNA identified by m^6^A-modified RNA immunoprecipitation sequencing (MeRIP-seq). Upper panel: MeRIP IP sample; lower panel: input control. **G** RIP-qPCR analysis showing PRRC2A enrichment on NCOA4 mRNA in TET cells, with values presented relative to IgG control. *n* = 5 in each group. Statistical analysis of the quantified data was performed using Student’s *t*-test. **H** Silver staining and Western blot analysis of PRRC2A in TET cells after RNA pull-down using either an NCOA4-specific or random probe. **I** Schematic representation of constructs used in dual-luciferase reporter assays. **J** Dual-luciferase reporter assay in Ty-82 cells with stable PRRC2A knockdown or control, assessing activity in cells transfected with wild-type (WT) or mutant (Mut) peak regions cloned into the GP-miRGLO vector. Relative firefly luciferase activity was normalized to Renilla luciferase and compared to the empty vector. *n* = 5 in each group. Statistical analysis of the quantified data was performed using Student’s *t*-test. **K** NCOA4 mRNA stability assay following actinomycin D treatment in Ty-82 cells. **L** Determination of NCOA4 mRNA half-life (t₁/₂) in Ty-82 cells after actinomycin D treatment. *n* = 5 in each group. Statistical significance was analyzed using two-way ANOVA for the decay curves and Student’s t-test for comparison of mRNA half-life. **M** Assessment of m^6^A mRNA methylation by dot blot analysis (m^6^A dot blot) in METTL3 knockdown and control Ty-82 and IU-TAB-1 cells. Methylene blue (MB) staining was used as a loading control. m^6^A quantification assay of METTL3 knockdown and control samples. **N** MeRIP-qPCR analysis was performed to examine the level of m6A in NCOA4 3′UTR in Ty-82 cells with or without knockdown of PRRC2A or METTL3. *n* = 5 in each group. Statistical analysis of the quantified data was performed using Student’s *t*-test. Western blot analysis of METTL3, PRRC2A, and NCOA4 protein expression in Ty-82 (**O**) and IU-TAB-1 (**P**) cells transfected with siMETTL3, shPRRC2A, or PRRC2A overexpression vector. **P < 0.01; ns not significant.

Based on the above results, we evaluated the expression levels of GABRD, NCOA4, RER1, and NIPAL3 in TET cells. RT-qPCR analysis revealed a consistent positive correlation between the mRNA levels of NCOA4, GABRD, RER1, and NIPAL3 and PRRC2A expression (Fig. 4A, B; Fig. S2A, B). Among these genes, NCOA4 demonstrated the most substantial changes in response to alterations in PRRC2A expression. Notably, NCOA4 has been recognized as playing a pivotal role in both metabolic regulation and ferroptosis [22, 26], consistent with our previous KEGG pathway enrichment analysis. Therefore, we subsequently focused our investigations on NCOA4. Western blot and immunofluorescence consistently demonstrated that PRRC2A positively regulates NCOA4 protein expression (Fig. 4C–E; Fig. S2C, D, F). IGV analysis of MeRIP-seq data revealed a significant enrichment of m^6^A peaks within the 3’UTR of NCOA4 mRNA, suggesting this region as a potential PRRC2A binding site (Fig. 4F). Given this observation, we hypothesized that PRRC2A may directly bind to NCOA4 through these m^6^A-modified regions. RIP-qPCR assays using anti-PRRC2A antibodies in TET cells. The results showed a marked enrichment of NCOA4 transcripts in the PRRC2A-immunoprecipitated fraction compared with the IgG control, demonstrating a specific PRRC2A-NCOA4 binding interaction (Fig. 4G; Fig. S2H). RNA pull-down assays were conducted using NCOA4 probes to directly capture interacting proteins. Silver staining and subsequent Western blot analysis of the pull-down complex revealed strong enrichment of PRRC2A, providing further evidence of a direct interaction between PRRC2A and NCOA4 mRNA (Fig. 4H).

To determine whether this interaction depends on the m^6^A sites within the NCOA4 3′UTR, we constructed dual-luciferase reporters carrying either wild-type (WT) or mutant (Mut) NCOA4 3′UTR sequences (Fig. 4I). Luciferase assays showed that PRRC2A knockdown significantly reduced FLuc/RLuc activity in the WT group, whereas no effect was observed in the Mut group (Fig. 4J). Considering previous reports suggesting PRRC2A modulates mRNA stability of target genes in an m^6^A-dependent manner [20, 21], we next performed Actinomycin D chase assays. PRRC2A knockdown markedly accelerated NCOA4 mRNA decay, indicating a reduced transcript half-life (Fig. 4K, L; Fig. S2E, G). Furthermore, to directly assess m^6^A dependence, we knocked down METTL3 in TET cells, which resulted in a significant reduction in global m^6^A levels (Fig. 4M). Consistently, MeRIP-qPCR analysis confirmed a marked decrease in m^6^A modification of NCOA4 transcripts following METTL3 depletion (Fig. 4N; Fig. S2I). Under these conditions, neither overexpression nor knockdown of PRRC2A affected NCOA4 mRNA abundance, indicating that the regulatory effect of PRRC2A on NCOA4 is strictly dependent on intact m^6^A modification (Fig. 4O, P; Fig. S2J).

Collectively, these results demonstrate that PRRC2A binds to m^6^A-modified sites within the NCOA4 3′UTR to stabilize NCOA4 mRNA.

### NCOA4 promotes glycolysis and malignant phenotypes in TET cells

**Fig. 5:**
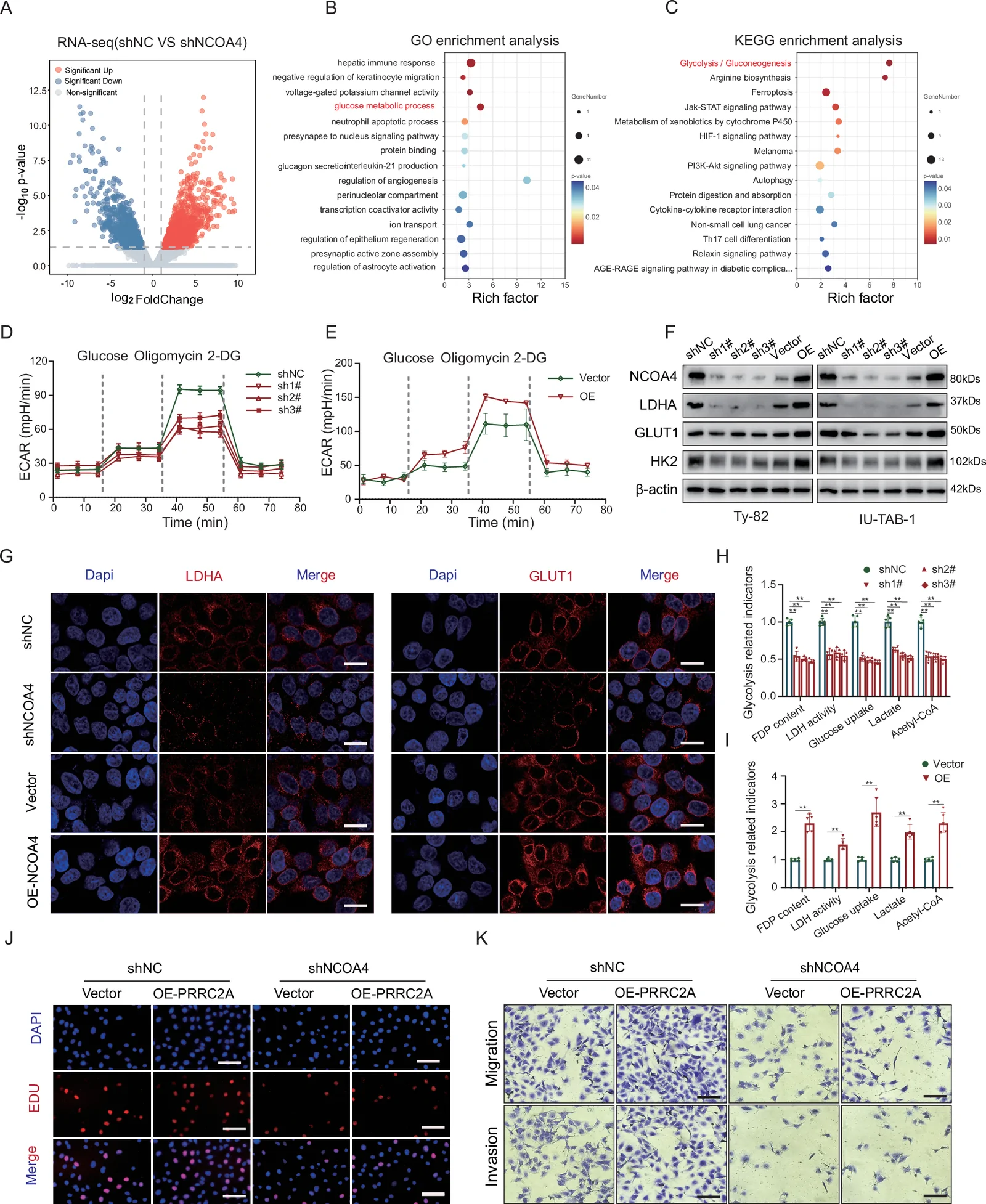
NCOA4 promotes glycolysis and malignant phenotypes in TET cells. **A** Volcano plot of differentially expressed genes of NCOA4 knockdown vs control Ty-82 cells. Enrichment analysis showing the biological process (**B**) and pathway enriched (**C**) following NCOA4 knockdown. The ECAR values of the Ty-82 cells with the knockdown (**D**) or overexpression (**E**) of NCOA4. **F** Western blotting analysis of NCOA4, LDHA, PKM2, GLUT1, and HK2 protein expression in Ty-82 and IU-TAB-1 cells transfected with the knockdown or overexpression of NCOA4. **G** Immunofluorescence (IF) was used to validate the changes in LDHA and GLUT1 protein levels (red fluorescent) in Ty-82 cells with knockdown or overexpression of NCOA4. (Scale bar: 10 μm). The FDP content, LDH activity, glucose uptake, lactate fold, and level of acetyl-CoA of the Ty-82 cells with the knockdown (**H**) or overexpression (**I**) of NCOA4. *n* = 5 in each group. Statistical analysis of the quantified data was performed using Student’s *t*-test. **J** EdU assays detecting the proliferation abilities of Ty-82 cells following shNCOA4 with or without PRRC2A overexpression vector transfection. (Scale bar: 50 μm). **K** Transwell migration and invasion assays in Ty-82 cells following shNCOA4 with or without PRRC2A overexpression vector transfection (Scale bar: 50 μm). **P < 0.01.

Given the identification of NCOA4 as a downstream effector of PRRC2A, we investigated NCOA4 biological role in TET cells. Ty-82, IU-TAB-1, and ThyL-6 cell lines with stable knockdown or overexpression of NCOA4 were established (Fig. S3A). RNA sequencing analysis of NCOA4-Knockdown TET cells revealed significant enrichment of metabolic pathways, particularly glycolysis and gluconeogenesis, in differentially expressed genes compared to control TET cells (Fig. 5A–C). To functionally validate these findings, glycolysis was assessed by measuring the extracellular acidification rate (ECAR) using a Seahorse analyzer. NCOA4 knockdown significantly reduced ECAR compared with control TET cells, whereas NCOA4 overexpression markedly increased ECAR (Fig. 5D, E; Fig. S3B–E). In addition, biochemical assays demonstrated that Knockdown of NCOA4 resulted in reduced glucose uptake, lactate production, fructose-1,6-bisphosphate content, lactate dehydrogenase (LDH) activity, and acetyl-CoA levels, whereas overexpression of NCOA4 led to significant increases in these glycolysis-related indicators (Fig. 5H, I; Fig. S3G). Next, we examined whether NCOA4 influences the expression of key glycolytic enzymes. Western blot analyses revealed that knockdown of NCOA4 significantly reduced the levels of LDHA, GLUT1, and HK2, while NCOA4 overexpression increased their expression (Fig. 5F; Fig. S3F). Immunofluorescence confirmed these alterations in glycolytic markers (Fig. 5G; Fig. S3H).

To determine whether NCOA4 mediates the effects of PRRC2A on TET cell behavior, we performed EdU and Transwell assays. NCOA4 knockdown significantly reduced the proliferative capacity of TET cells, as indicated by decreased EdU-positive cells, and markedly inhibited cell migration and invasion. Moreover, when PRRC2A was overexpressed in TET cells with NCOA4 knockdown, the PRRC2A-induced enhancement of proliferation, migration, and invasion was largely abolished (Fig. 5J, K; Fig. S3I–L), indicating that NCOA4 serves as a critical downstream effector through which PRRC2A promotes tumor progression.

Collectively, these data indicate that NCOA4 promotes glycolytic reprogramming and enhances the proliferative and invasive capacities of TET cells.

### NCOA4 directly interacts with PKM2 and drives glycolysis regulation in TET cells

**Fig. 6:**
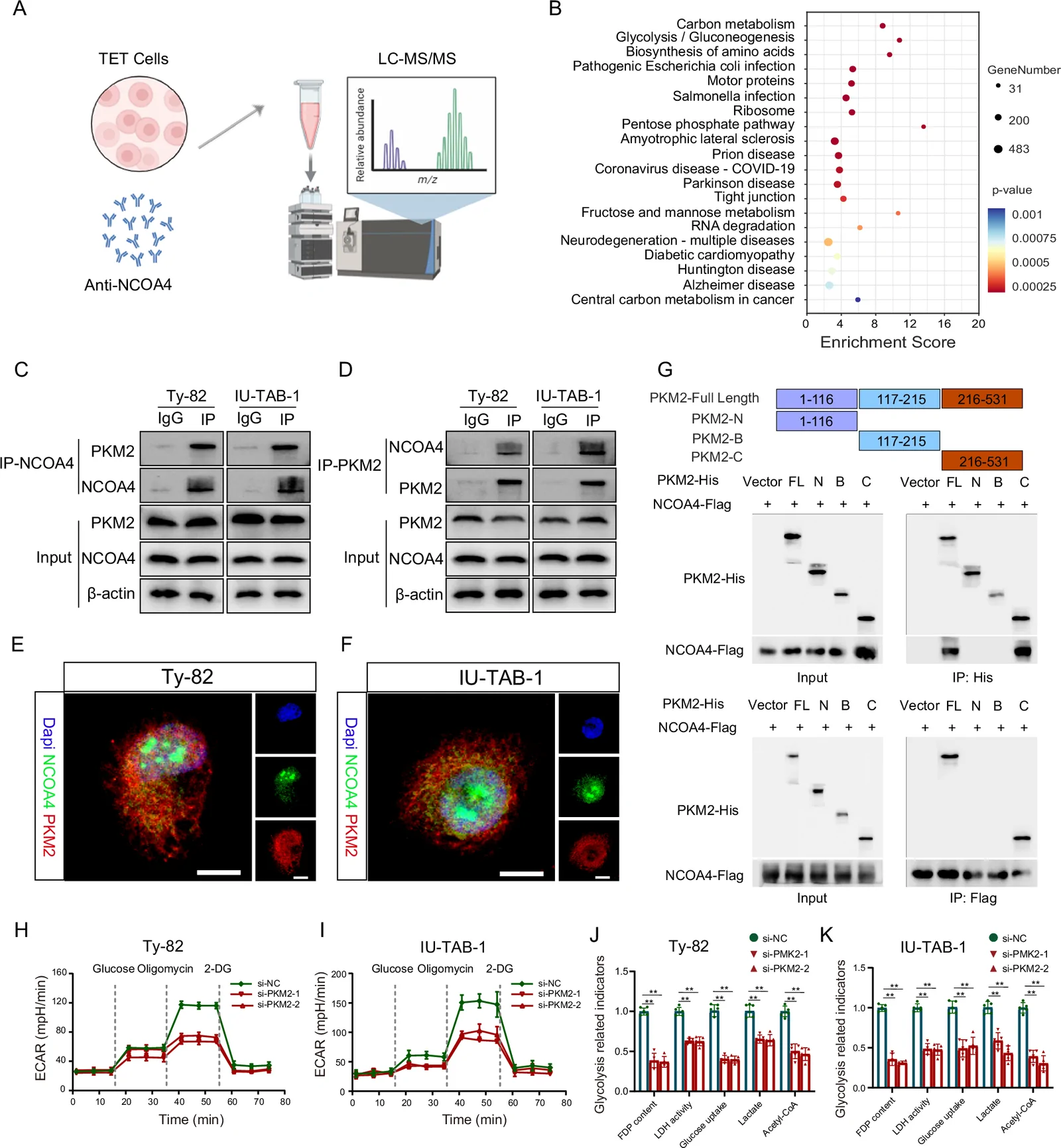
NCOA4 directly interacts with PKM2 and drives glycolysis regulation in TET cells. **A** 2 × 10^7^ TET cells were collected, and then a co-IP assay was performed with anti-NCOA4 antibody to obtain proteins that directly interact with NCOA4. Subsequently, the proteins were identified by mass spectrometry. **B** Enrichment analysis showing the pathway enriched in the NCOA4 combined protein. **C**, **D** The direct interaction between NCOA4 and PKM2 was revealed by co-IP assays and analyzed by western blot assays. Representative immunofluorescence images showing the cellular localization of NCOA4 and PKM2 in Ty-82 (**E**) and IU-TAB-1 (**F**) cells. (Scale bar: 3 μm). **G** The His-tagged PKM2 full-length and fragment plasmid (top) and Flag-tagged NCOA4 overexpression plasmid were constructed. The Flag-tagged NCOA4 and His-tagged PKM2 overexpression plasmid were simultaneously transfected into 293T cells, and co-IP analysis was then performed to detect the domain PKM2 interacted with NCOA4 using anti-His tag (middle) and anti-Flag tag antibodies (bottom). The ECAR values of the Ty-82 (**H**) and IU-TAB-1 (**I**) cells with the knockdown of PKM2. The FDP content, LDH activity, glucose uptake, lactate fold, and level of acetyl-CoA of the Ty-82 (**J**) and IU-TAB-1 (**K**) cells with the knockdown of PKM2. *n* = 5 in each group. Statistical analysis of the quantified data was performed using Student’s *t*-test. **P < 0.01.

To illustrate the regulatory effects of NCOA4 on TET cells' progression from a molecular mechanism perspective, we aimed to identify the proteins that interact with NCOA4. Co-immunoprecipitation (Co-IP) combined with mass spectrometry analysis identified a series of NCOA4-interacting proteins (Fig. 6A). Functional enrichment analysis indicated that these proteins were primarily involved in carbon metabolism, glycolysis, various biosynthetic pathways, as well as other metabolic processes (Fig. 6B). Notably, PKM2 exhibited the highest enrichment score among the NCOA4-interacting proteins identified by mass spectrometry (Table S5). Reciprocal Co-IP confirmed this interaction in TET cells (Fig. 6C, D; Fig. S4A). Immunofluorescence analysis further demonstrated cytoplasmic co-localization of NCOA4 and PKM2 (Fig. 6E, F; Fig. S4B). Furthermore, domain-mapping experiments revealed that the C-terminal region of PKM2 mediates its interaction with NCOA4 (Fig. 6G). Functional assays indicated that silencing PKM2 significantly reduced glycolytic activity, as evidenced by decreased ECAR, and abrogated NCOA4-induced glycolytic enhancement (Fig. 6H–K; Fig. S4C, D).

These results suggest that NCOA4 directly interacts with PKM2 and drives glycolysis regulation in TET cells.

### PKM2 was a key downstream molecule of NCOA4, promoting TET progression

**Fig. 7:**
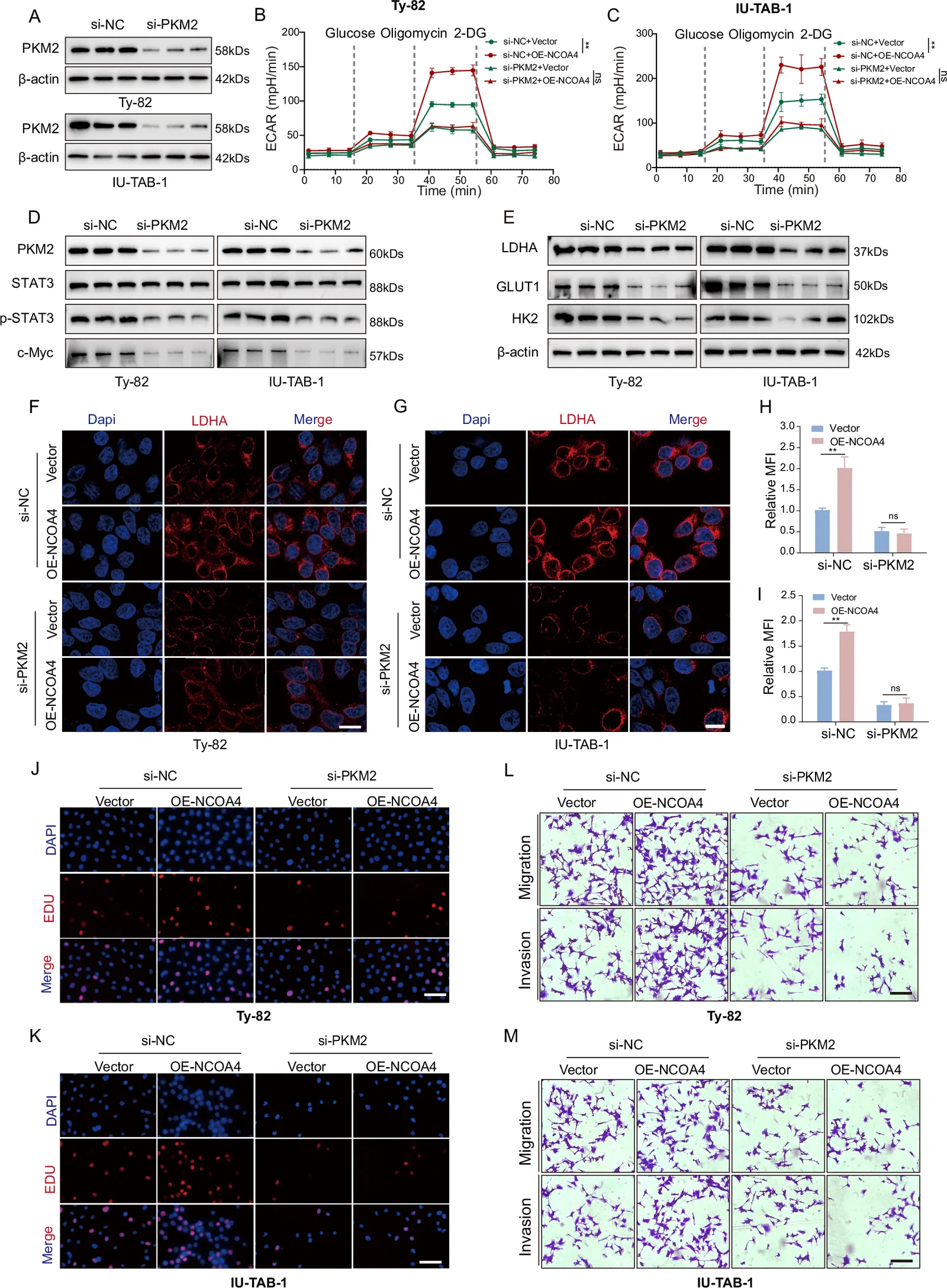
PKM2 was a key downstream molecule of NCOA4, promoting TET progression. **A** Western blot analysis of PKM2 protein levels in TET cells following knockdown. Measurement of extracellular acidification rate (ECAR) in Ty-82 (**B**) and IU-TAB-1 (**C**) cells transfected with siPKM2 with or without NCOA4 overexpression vector. **D**, **E** Western blot analysis of PKM2, STAT3, phosphorylated STAT3 (p-STAT3), c-Myc, LDHA, GLUT1, and HK2 protein levels in TET cells transfected with siNC or siPKM2. Immunofluorescence (IF) analysis of LDHA and GLUT1 protein expression (red fluorescence) in Ty-82 (**F**, **H**) and IU-TAB-1 (**G**, **I**) cells transfected with siPKM2 with or without NCOA4 overexpression vector (Scale bar: 10 μm). *n* = 5 in each group. Statistical analysis of the quantified data was performed using Student’s *t*-test. EdU incorporation assays assessing the proliferative capacity of Ty-82 (**J**) and IU-TAB-1 (**K**) cells with siPKM2 with or without NCOA4 overexpression vector (Scale bar: 50 μm). Transwell migration and invasion assays in Ty-82 (**L**) and IU-TAB-1 (**M**) cells following siPKM2 with or without NCOA4 overexpression vector transfection (Scale bar: 50 μm). **P < 0.01; ns not significant.

To verify whether NCOA4 promotes glycolysis and progression of TET via PKM2, we knocked down PKM2 expression in TET cells (Fig. 7A; Fig. S5A). We assessed the glycolytic capacity of TET cells overexpressing NCOA4 in the presence or absence of PKM2 knockdown. Results demonstrated that PKM2 knockdown suppressed NCOA4-induced enhancement of extracellular acidification rate and glycolysis-related indicators in TET cells (Fig. 7B, C; Fig. S5B, F–H), suggesting PKM2 as a crucial mediator of NCOA4-driven glycolysis. Given that PKM2 regulates glycolysis directly through catalytic activity and indirectly through its nuclear role as a transcriptional regulator in the STAT3-c-Myc signaling pathway, thereby promoting the expression of glycolysis-related genes such as LDHA, GLUT2, and HK2 [32, 33]. We next examined the expression of these markers. Results showed that PKM2 knockdown significantly reduced phosphorylation of STAT3 and decreased the expression levels of c-Myc, LDHA, GLUT2, and HK2 (Fig. 7D, E; Fig. S5C). Immunofluorescence (IF) analysis also indicated that PKM2 knockdown inhibited the NCOA4-induced upregulation of glycolytic markers LDHA and GULT1 in TET cells (Fig. 7F–I; Fig. S5D). Next, we investigated the effects of glycolysis inhibition through PKM2 knockdown on TET proliferation and metastasis. EDU assays further confirmed these findings (Fig. 7J, K; Fig. S5E, J). Our previous findings demonstrated that NCOA4 overexpression promotes migration and invasion in TET cells; notably, these promoting effects were effectively reversed by simultaneous knockdown of PKM2 (Fig. 7L, M; Fig. S5I, K, L).

Taken together, these results indicate that PKM2 functions as a pivotal downstream mediator of NCOA4, facilitating glycolysis and thereby promoting TET progression.

### Combined inhibition of PRRC2A and NCOA4 synergistically suppresses TET progression in vivo

**Fig. 8:**
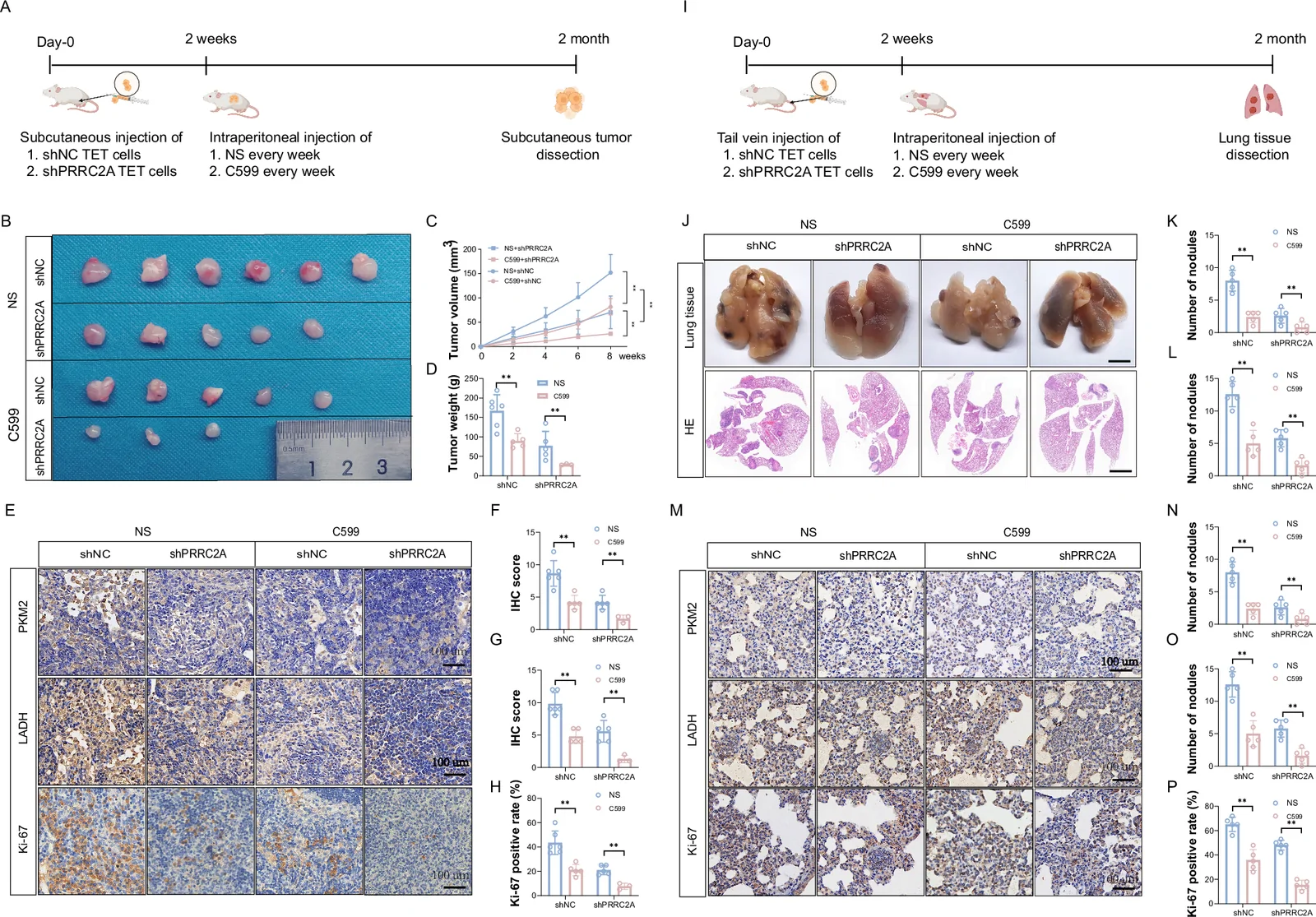
Combined inhibition of PRRC2A and NCOA4 synergistically suppresses TET progression in vivo. **A** Representative images of subcutaneous xenograft tumors in BALB/c nude mice injected with Ty-82 cells with or without C599 treatment. **B**–**D** Quantification of subcutaneous xenograft tumor volume and weight in BALB/c nude mice injected with Ty-82 cells with or without C599. *n* = 6 mice per group. Tumor growth curves were analyzed using two-way repeated-measures ANOVA, and endpoint tumor weights were analyzed using Student’s *t*-test. **E** Immunohistochemical staining of GLUT1, LDHA, and Ki-67 in subcutaneous xenograft tumors from BALB/c nude mice injected with Ty-82 cells with or without C599 treatment (Scale bar: 100 μm). Quantitative analysis of immunohistochemistry (IHC) staining scores for GLUT1 (**F**), LDHA (**G**), and Ki-67 positivity (**H**) in subcutaneous xenograft tumors from BALB/c nude mice injected with Ty-82 cells with or without C599. *n* = 5 in each group. Statistical analysis of the quantified data was performed using Student’s *t*-test. **I** Representative images of lung tissues from BALB/c nude mice injected via the tail vein with Ty-82 cells with or without C599 treatment. **J** Representative images of excised lung tissues and corresponding H&E-stained sections. Quantification of metastatic nodules in lung tissues (**K**) and H&E sections (**L**) from mice injected with Ty-82 cells with or without C599. *n* = 5 in each group. Statistical analysis of the quantified data was performed using Student’s *t*-test. **M** Immunohistochemical staining of GLUT1, LDHA, and Ki-67 in lung tissues from BALB/c nude mice injected via the tail vein with Ty-82 cells with or without C599 (Scale bar: 100 μm). Quantitative analysis of IHC staining scores for GLUT1 (**N**), LDHA (**O**), and Ki-67 positivity (**P**) in lung tissues from BALB/c nude mice injected via tail vein with Ty-82 cells with or without C599 (Scale bar: 100 μm). *n* = 5 in each group. Statistical analysis of the quantified data was performed using Student’s *t*-test. **P < 0.01.

Given that PRRC2A stabilizes NCOA4 mRNA and that NCOA4 drives glycolytic activation via PKM2, we hypothesized that simultaneous targeting of the PRRC2A-NCOA4-PKM2 axis could exert synergistic antitumor effects. To test this, we evaluated the efficacy of C599, a pharmacological inhibitor of PKM2, alone or in combination with PRRC2A knockdown in xenograft models (Fig. 8A). Treatment with C599 significantly reduced tumor growth and weight compared with normal saline (NS), importantly, the combination of C599 with PRRC2A silencing resulted in further suppression of tumor volume and weight compared with either intervention alone (Fig. 8B–D). In addition, C599 treatment decreased the protein expression levels of GLUT1 and LDHA in xenograft tumors, and the combination of C599 with PRRC2A knockdown resulted in even greater suppression of these representative glycolytic proteins (Fig. 8E–G). Immunohistochemical analysis revealed that PRRC2A knockdown reduced the number of Ki-67-positive cells in TET tumor tissues, and this inhibitory effect was further enhanced by combined treatment with C599 (Fig. 8H).

Furthermore, we investigated whether the regulatory effect of PRRC2A on C599 impacts TET cell metastasis in vivo (Fig. 8I). Lung metastasis assays demonstrated that PRRC2A knockdown significantly decreased the number of metastatic nodules, whereas the combination with C599 further suppressed TET lung metastasis (Fig. 8J–L). In addition, C599 treatment decreased the protein expression levels of GLUT1 and LDHA in metastatic nodules, and the combination of C599 with PRRC2A knockdown resulted in even greater suppression of these representative glycolytic proteins (Fig. 8M–O). Immunohistochemical analysis revealed that PRRC2A knockdown reduced the number of Ki-67 positive cells in TET tumor tissues, and this inhibitory effect was further enhanced by combined treatment with C599 (Fig. 8M, P).

Collectively, our findings confirm the therapeutic potential of C599 in TET and indicate that low expression of PRRC2A can further augment the efficacy of C599 treatment. These results provide a promising therapeutic strategy for TET.

### PRRC2A expression positively correlates with NCOA4 and PKM2 in TET tissues

**Fig. 9:**
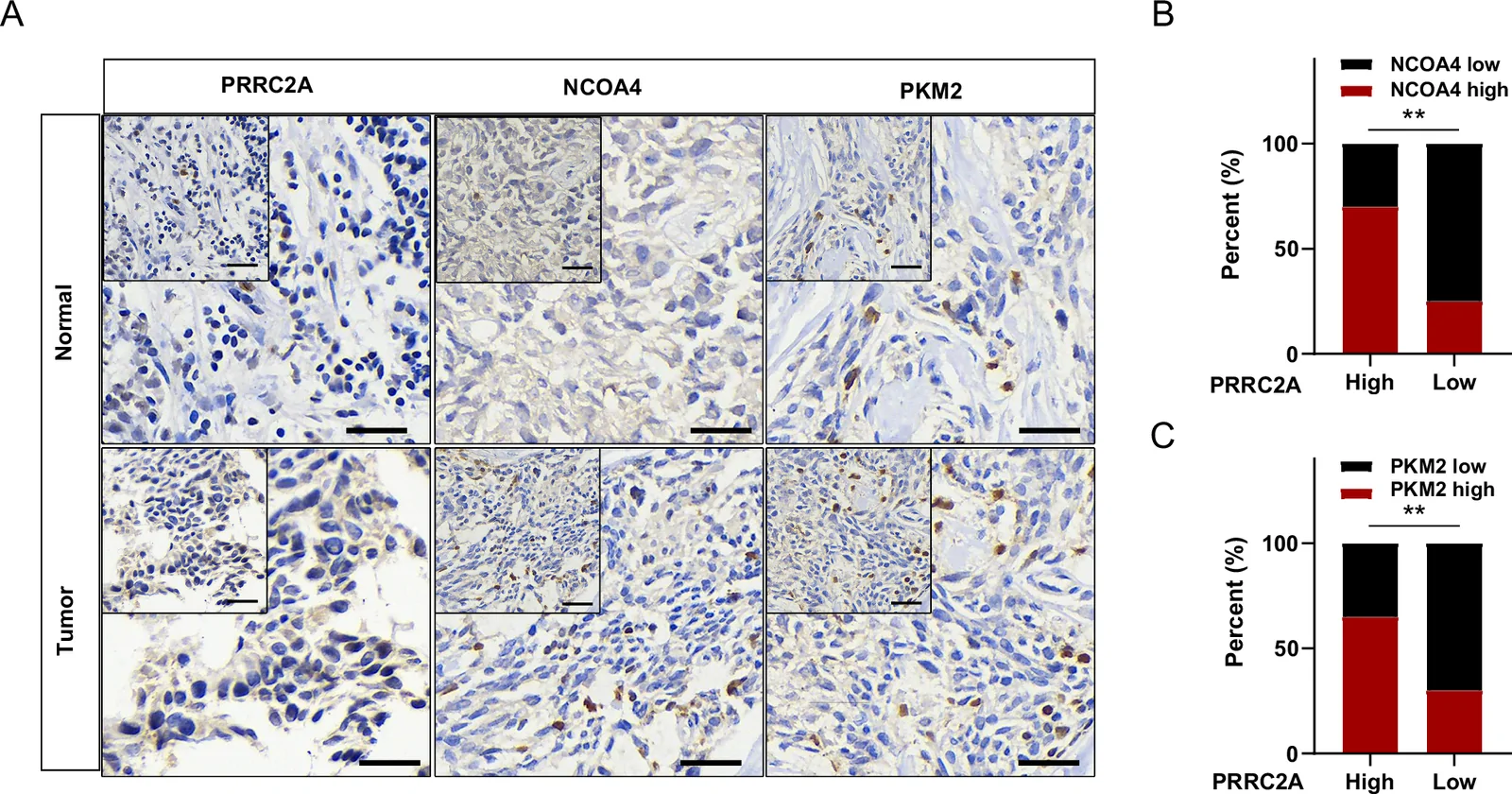
PRRC2A expression positively correlates with NCOA4 and PKM2 in TET tissues. **A** Representative IHC images of PRRC2A, NCOA4, and PKM2 expression in 70 TET tissues. Scale bar: 50 μm. **B**, **C** Correlation analysis between PRRC2A and NCOA4 or PKM2 protein expression levels, as determined by the chi-square test (*n* = 70).

We further analyzed the correlations of PPRRC2A with NCOA4 and PKM2 using our TET samples. As expected, a positive correlation between the PRRC2A levels and NCOA4 or PKM2 protein expression. Specifically, 68.89% and 64.44% of the TET tissues with high PRRC2A expression exhibited high NCOA4 and PKM2 protein expression, respectively. Moreover, 76% and 72% of TETs tissues with low PRRC2A levels presented low levels of NCOA4 and PKM2 protein expression (Fig. 9A–C).

Collectively, these findings indicate that the PRRC2A-NCOA4-PKM2 axis is coordinately upregulated in TET tissues and may play an important role in TET progression.

## Discussion

Although the incidence of TETs has remained stable or increased in recent years, mortality rates have declined, likely as a result of improvements in surgical techniques and pharmacological treatments [34]. Recent clinical trials have demonstrated that the tyrosine kinase inhibitors sunitinib and lenvatinib possess potential efficacy in patients with chemotherapy-refractory TET. However, overall response rates to these agents remain suboptimal [35, 36]. With regard to immune checkpoint inhibitors (ICIs), pembrolizumab and nivolumab have been utilized as non-first-line therapies for TETs [37, 38]. Nonetheless, only a small subset of patients achieves partial responses, and the frequency of immune-related adverse events remains relatively high. These limitations underscore the urgent need to clarify the molecular mechanisms underlying TET pathogenesis to facilitate the development of more effective therapeutic strategies. In our study, we identified a novel mechanism in which PRRC2A upregulates NCOA4 expression in an m^6^A-dependent manner, thereby establishing a unique NCOA4-PKM2 regulatory axis that drives glycolytic reprogramming in TETs. This discovery provides a new perspective for metabolic targeting in TETs and holds significant translational potential.

Recent studies have increasingly implicated dysregulated RNA modifications, particularly N6-methyladenosine (m^6^A), in the pathogenesis of TETs [11, 13]. Aberrant expression of m^6^A methyltransferases (such as METTL3) or demethylases (such as FTO and ALKBH5) can alter the modification status of transcripts encoding oncogenes or tumor suppressor genes, thus promoting tumor cell proliferation, invasion, and metastasis [39–41]. However, compared with writers and erasers, the functional roles of m6A “reader” proteins in TETs remain poorly characterized.

PRRC2A is a recently identified m^6^A reader protein that was initially described as a regulator of oligodendrocyte specification and myelination [20]. In cancer biology, PRRC2A has been shown to promote colorectal cancer progression via activation of the CK1ε-mediated WNT and YAP signaling pathways [21]. Our findings extend these observations by demonstrating that PRRC2A is also upregulated in TETs and is significantly associated with adverse clinicopathological features and unfavorable prognosis. These results suggest that PRRC2A may function as a broadly relevant oncogenic m6A reader across multiple tumor types, rather than exerting tissue-restricted effects.

Mechanistically, prior studies have indicated that PRRC2A recognizes m6A-modified transcripts via a conserved GGACU motif, thereby enhancing translation efficiency or stabilizing target mRNAs such as Olig2 [19, 20]. Consistent with these reports, our integrated MeRIP-seq, bulk RNA-seq, and RIP-seq analyses identify NCOA4 as a direct downstream effector of PRRC2A in TETs. Functional assays further demonstrate that PRRC2A stabilizes NCOA4 mRNA in an m6A-dependent manner, reinforcing the concept that PRRC2A primarily acts by regulating transcript stability within the epitranscriptomic network.

NCOA4 is well recognized for its role in mediating ferritinophagy and maintaining intracellular iron homeostasis [24]. More recently, studies have shown that NCOA4 can also regulate metabolic enzyme activity through non-ferritinophagy pathways in malignancies such as ovarian and liver cancer [26, 27]. In our study, immunoprecipitation-mass spectrometry, co-immunoprecipitation, and rescue experiments revealed for the first time a direct interaction between NCOA4 and PKM2, thus establishing an NCOA4-PKM2 regulatory axis and providing new insights into the metabolic functions of NCOA4 in cancer.

Tumor cells depend on aerobic glycolysis (the Warburg effect) to generate biosynthetic precursors that support rapid proliferation [42]. In TETs, key glycolytic transcription factors or enzymes, including c-Myc and GLUT1, are overexpressed [43, 44]; however, the upstream regulatory mechanisms governing their expression have not been fully elucidated. Our study demonstrates that interaction between NCOA4 and PKM2 promotes glycolytic activity in TET cells, whereas PKM2 knockdown abolishes the glycolytic effects mediated by NCOA4. These findings indicate that PKM2 is a critical downstream mediator of NCOA4. PKM2 not only directly promotes lactate production but also translocates to the nucleus, where it facilitates STAT3 phosphorylation and c-Myc expression, thereby upregulating GLUT1, LDHA, and HK2 and further enhancing glycolysis, promoting the Warburg effect, and driving TET cell proliferation and metastasis.

From a therapeutic perspective, PKM2 has attracted increasing attention as a druggable metabolic target. The small-molecule PKM2 inhibitor C599 has previously demonstrated antitumor activity in aggressive glioblastoma models. Here, we extend these findings by showing that C599 effectively suppresses glycolysis, tumor growth, and metastasis in TET models in vivo. Importantly, combined PKM2 inhibition and PRRC2A knockdown produced synergistic antitumor effects, underscoring the therapeutic potential of targeting the PRRC2A-NCOA4-PKM2 axis.

Several limitations of this study warrant consideration. A substantial proportion of the mechanistic and in vivo experiments were performed using the Ty-82 cell line, which was originally derived from a thymic carcinoma but was subsequently reclassified as a thoracic NUT carcinoma based on the presence of a BRD4-NUT fusion, according to the 2021 WHO classification. Given that NUT carcinoma represents a rare, molecularly defined subtype within thymic epithelial malignancies, its biological behavior may not fully recapitulate that of conventional thymomas or thymic carcinomas. To mitigate this limitation, we validated the core regulatory features of the PRRC2A-NCOA4-PKM2 axis in additional TET cell lines, including IU-TAB-1 and ThyL-6, where consistent effects on glycolysis and tumor-associated phenotypes were observed. Nonetheless, further validation in larger patient cohorts and additional experimental systems will be required to establish the broader clinical relevance of these findings.

In conclusion, our findings demonstrate that PRRC2A enhances the stability of NCOA4 mRNA through recognition of m^6^A modifications, leading to the accumulation of NCOA4 protein. NCOA4 directly binds PKM2 and facilitates its nuclear translocation, thereby promoting STAT3 phosphorylation, increasing glycolytic flux, and enhancing the Warburg effect, ultimately facilitating TET proliferation and metastasis. Furthermore, the PKM2 inhibitor C599 significantly suppresses the progression of TET. Knockdown of PRRC2A further enhances the inhibitory effect of C599 on TET cells. This regulatory axis not only elucidates a novel mechanism underlying TET tumorigenesis and progression but also provides new potential targets for therapeutic intervention.

## Supplementary information

Supplementary Materials

Original Western blots

## Data Availability

The single-cell RNA-seq datasets analyzed in this study are publicly available in the Gene Expression Omnibus (GEO) under accession number GSE147520 and in the National Genomics Data Center under accession number HRA002334. All data supporting the findings of this study are available within the main text and Supplementary Materials. The sequencing raw data generated in this study are available from the corresponding author upon reasonable request.
