# Supplementary material for: PRRC2A-mediated m6A modification on NCOA4 promotes thymic epithelial tumor progression through PKM2-induced glycolysis
Source: Cell Death Dis. 2026 Jul 8;17(1):851. doi: 10.1038/s41419-026-08883-y (PMC13631309; doi:10.1038/s41419-026-08883-y)

Figure 1L

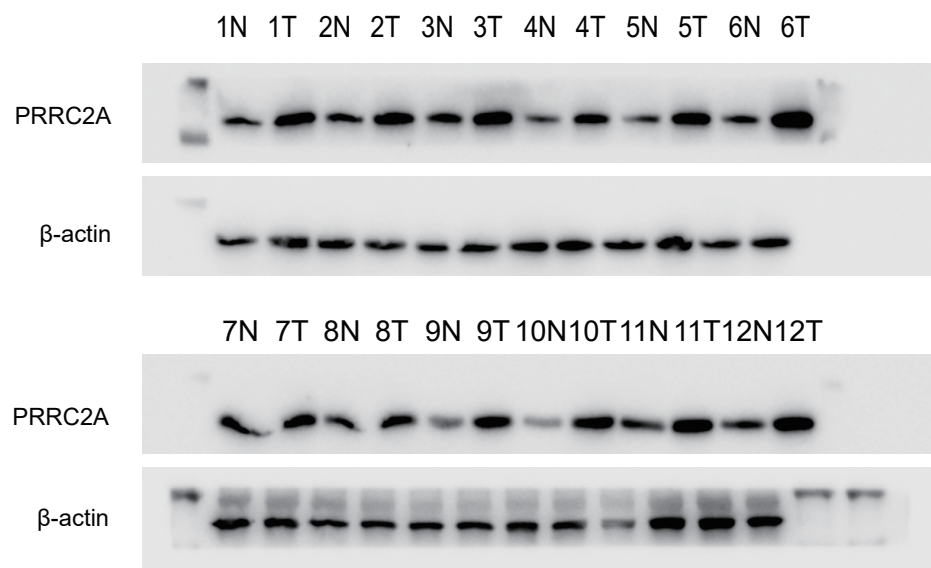

Figure 4C

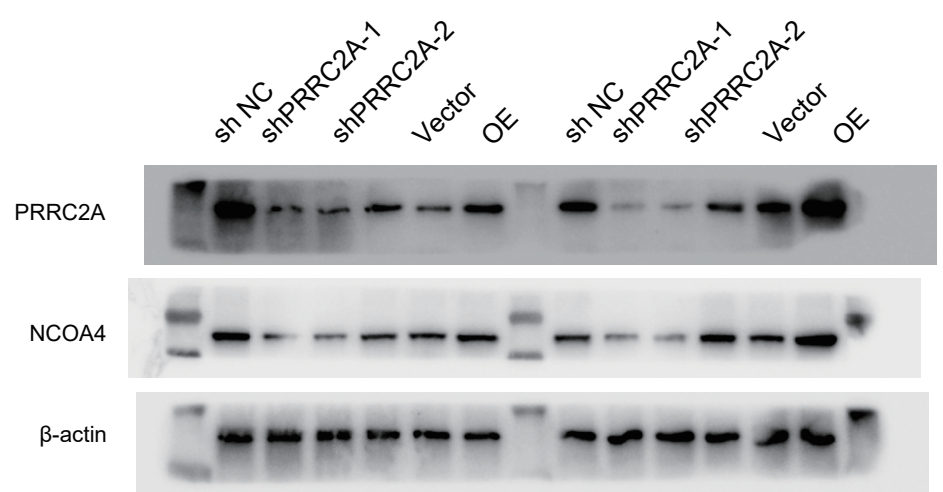

Figure 4H

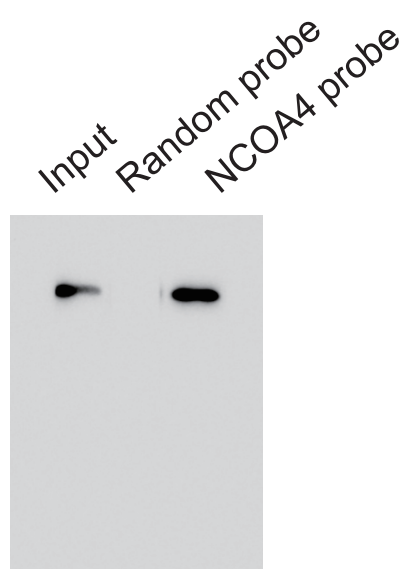

Figure 4M

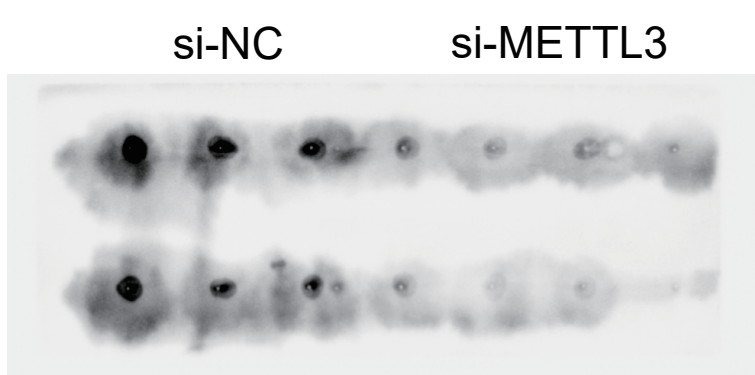

Figure 4O,P

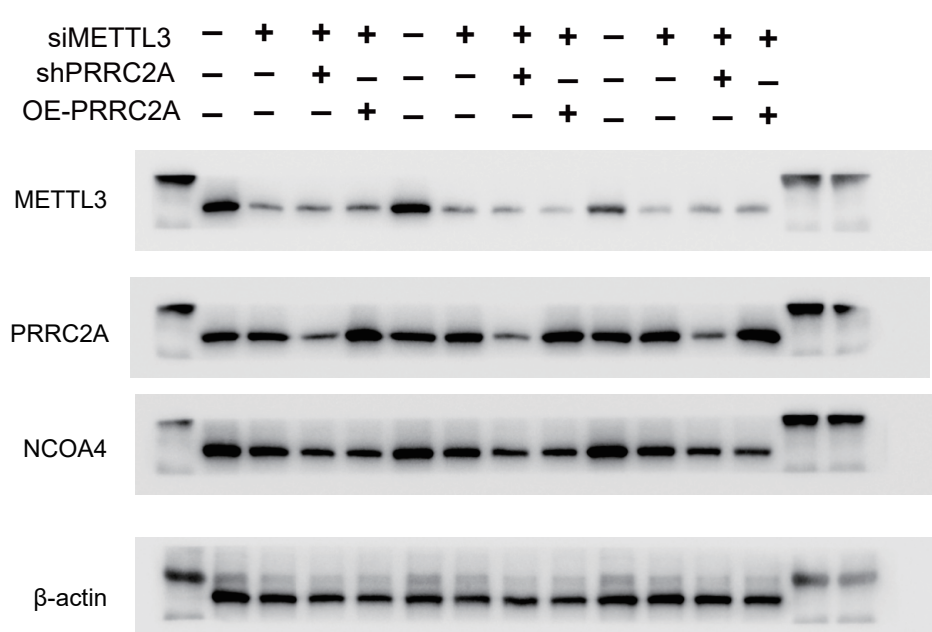

Figure 5F

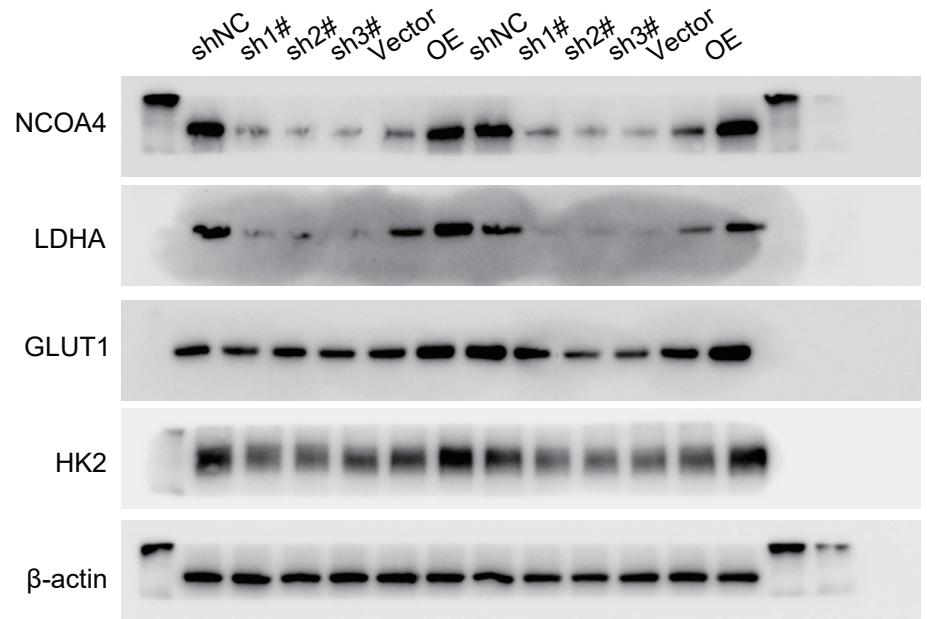

Figure 6C,D

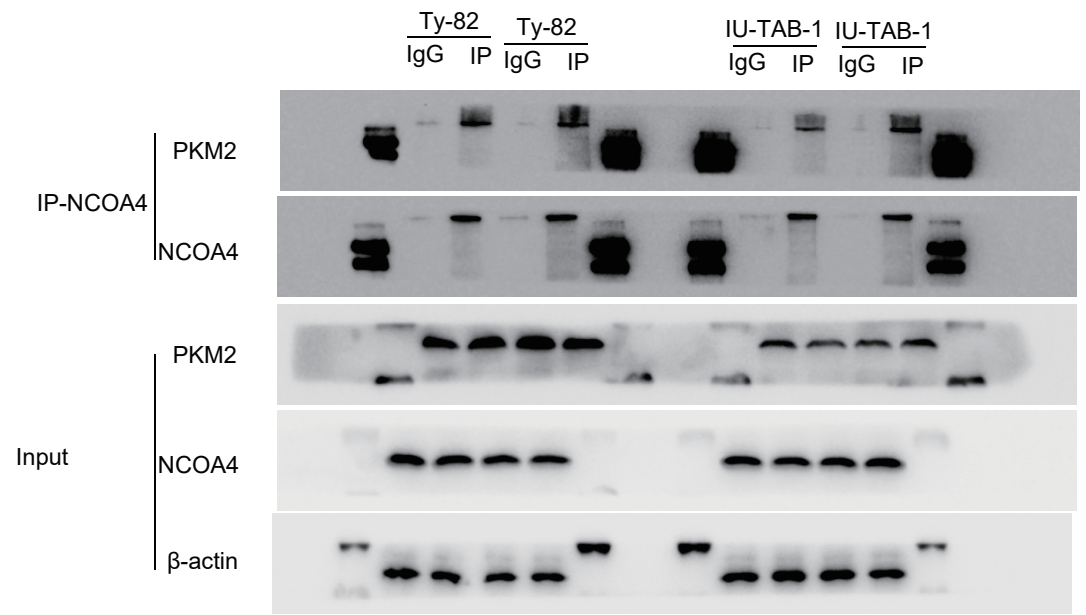

Figure 6G

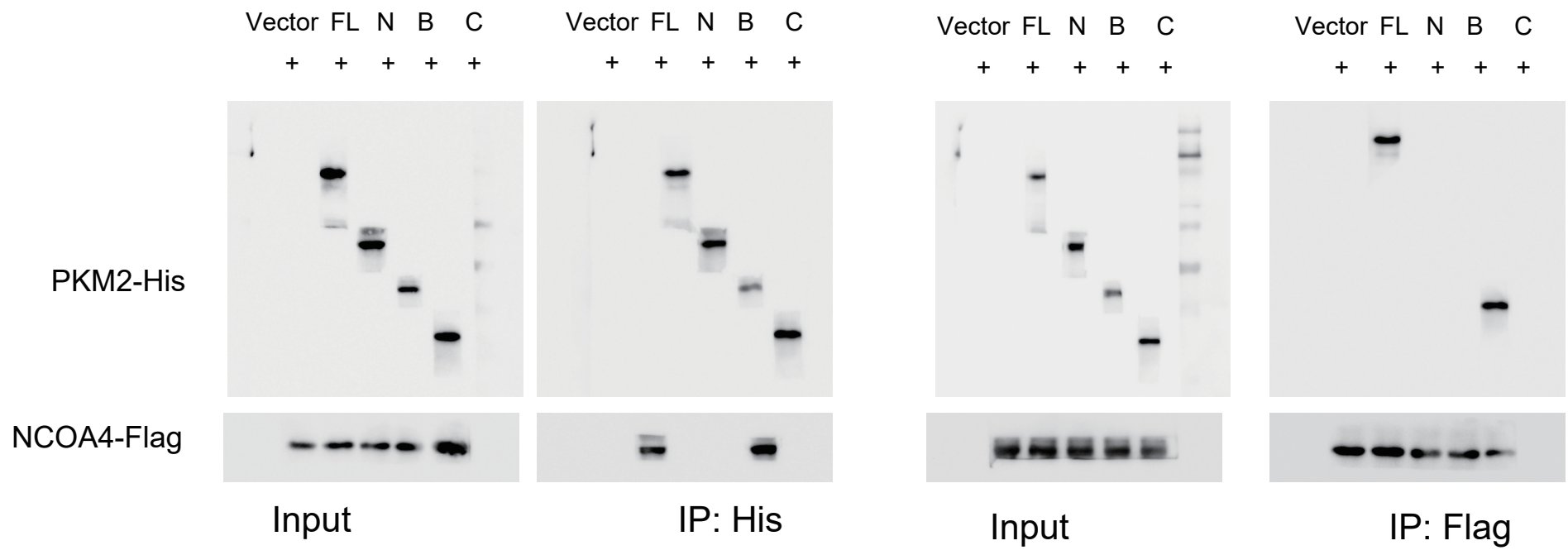

Figure 7A

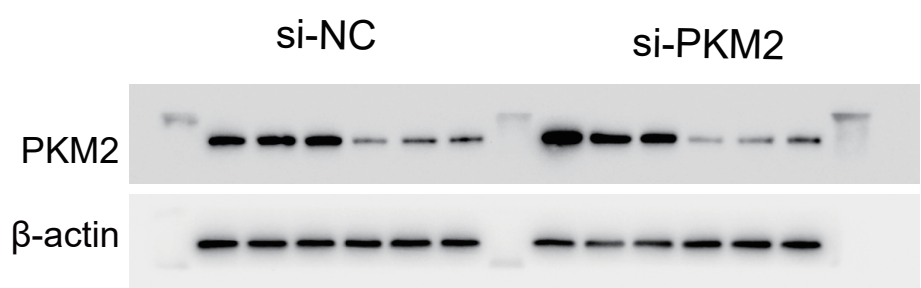

Figure 7D

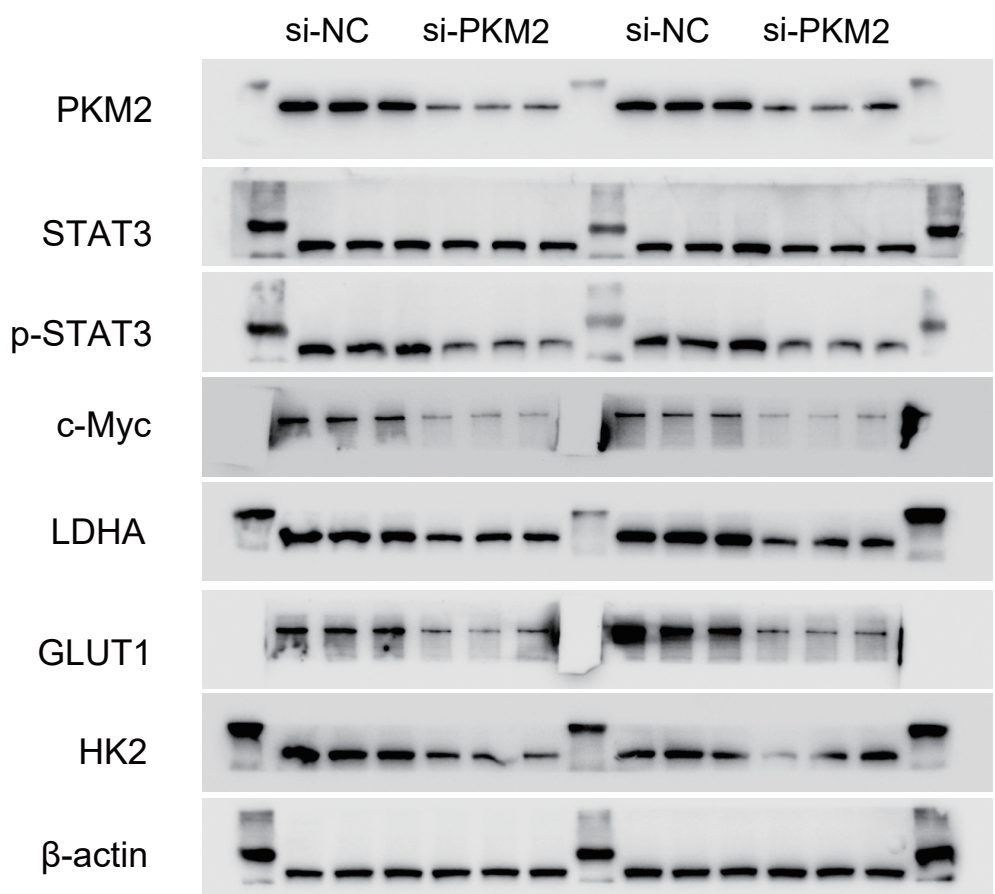

Figure S1A

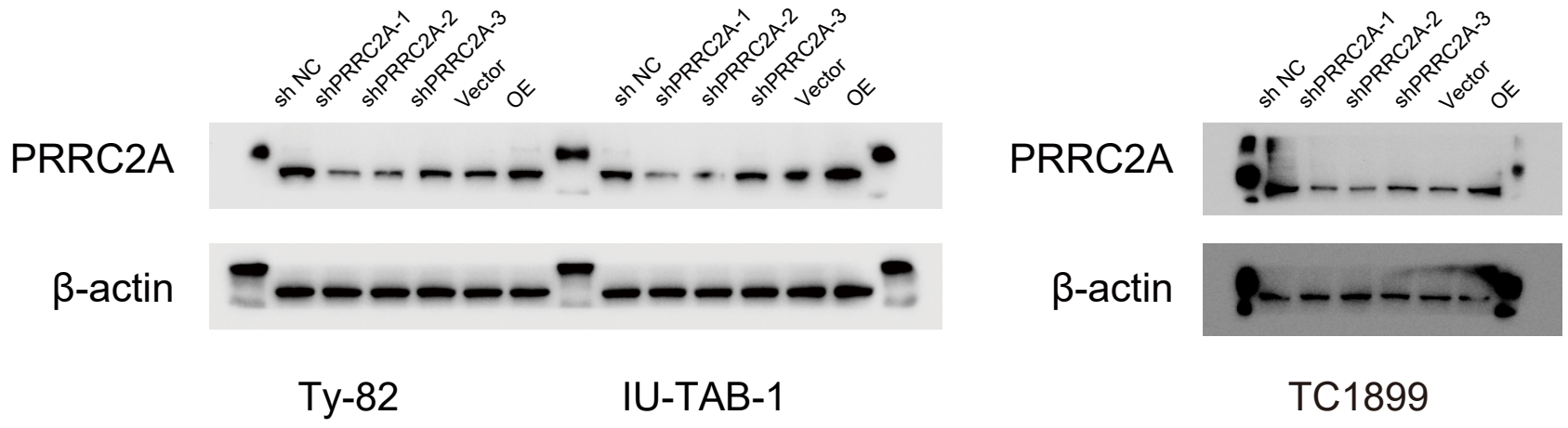

Figure S2C

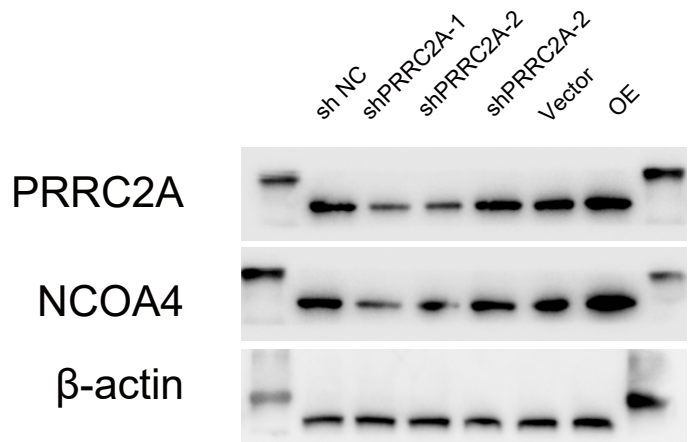

Figure S2J

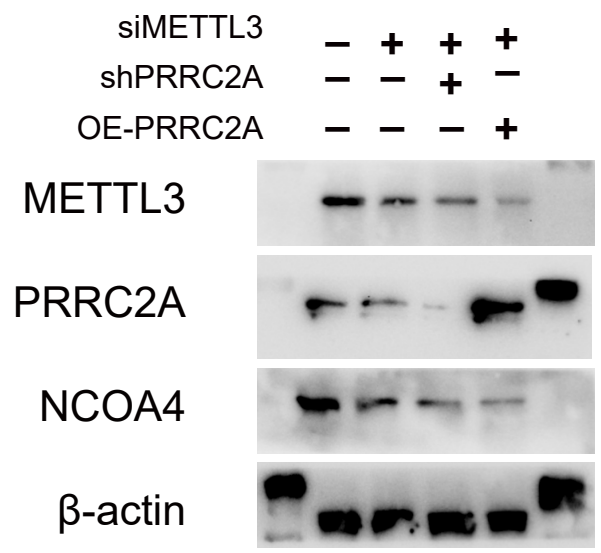

Figure S3A

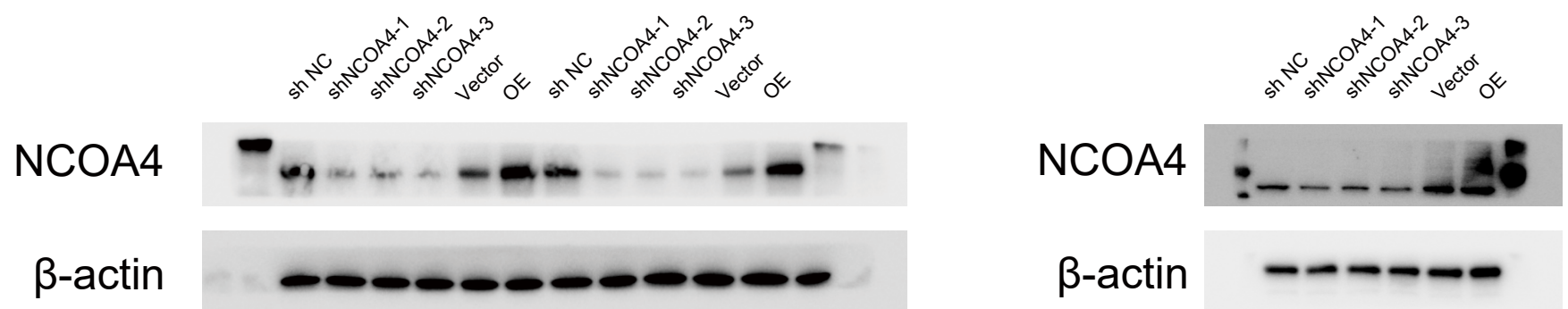

Figure S3F

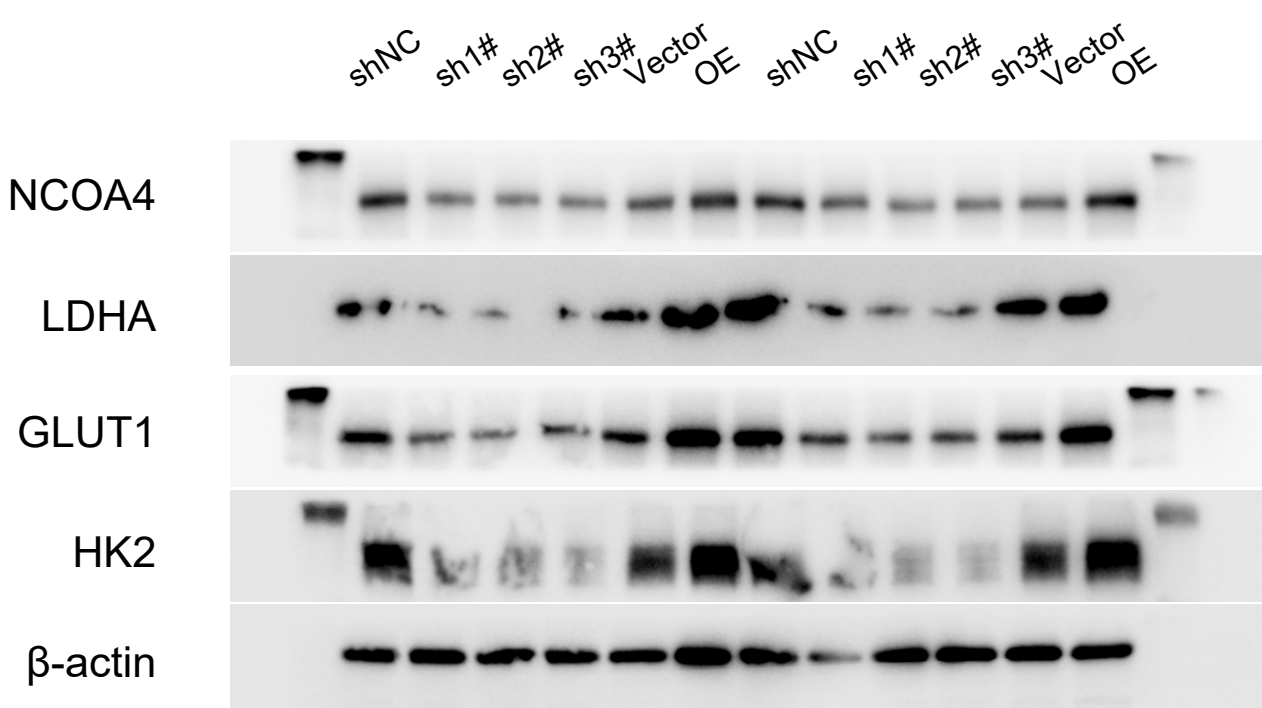

Figure S4A

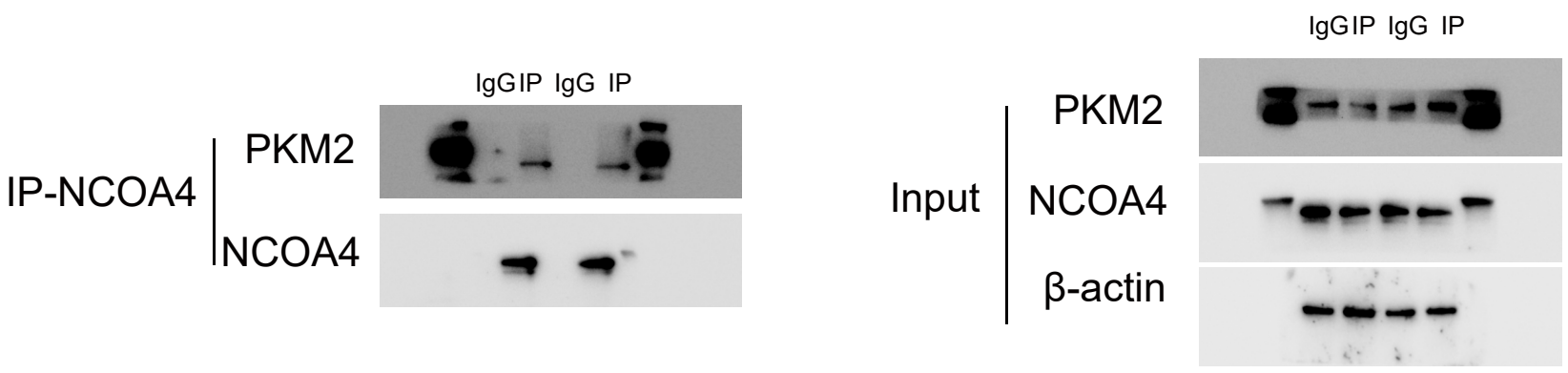

Figure S5A

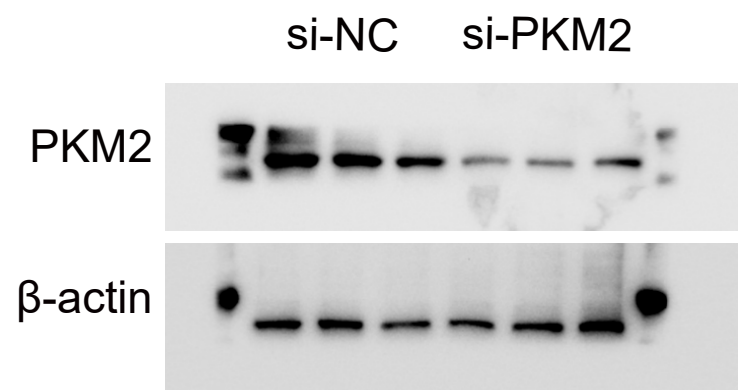

Figure S5C

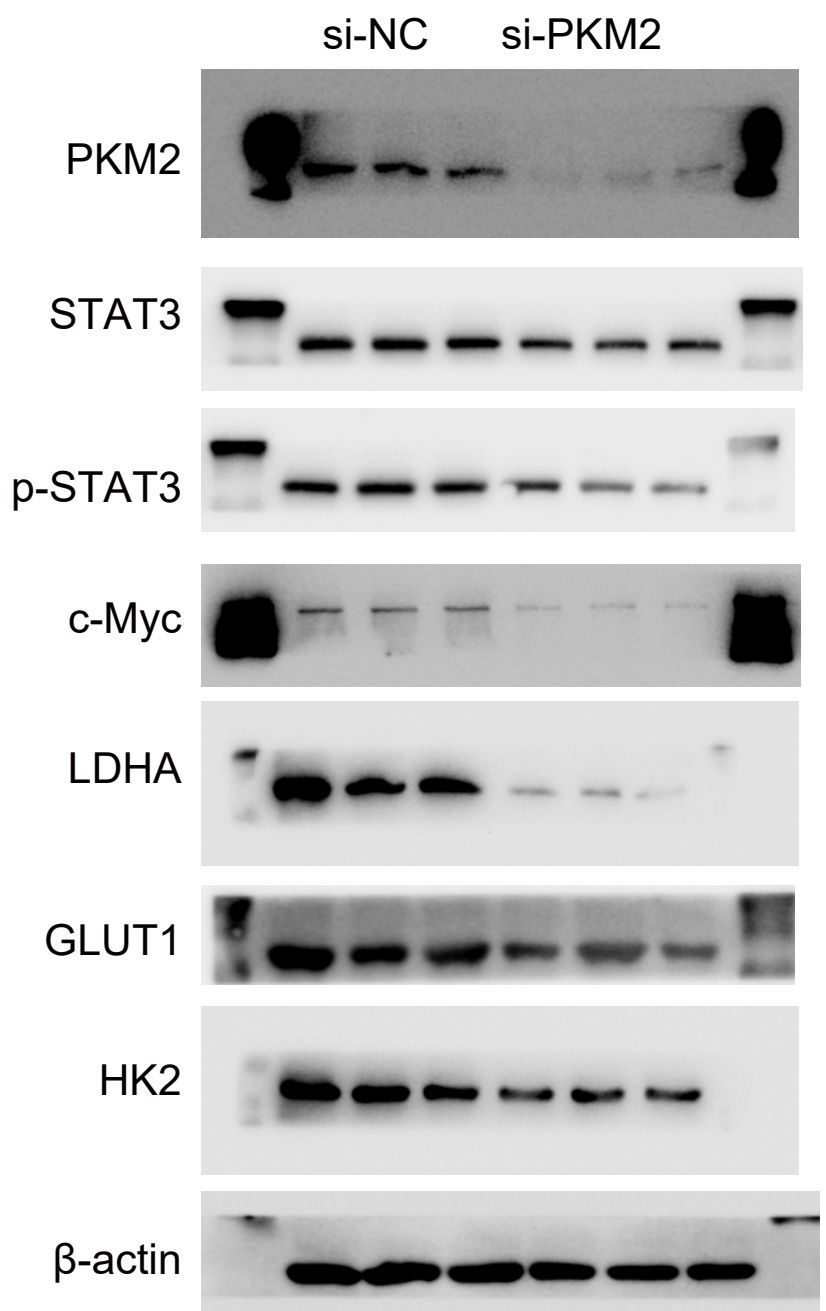

Supplement: Supplementary file 1 [file 41419_2026_8883_MOESM2_ESM.pdf]
