# Supplementary material for: PRRC2A-mediated m6A modification on NCOA4 promotes thymic epithelial tumor progression through PKM2-induced glycolysis
Source: Cell Death Dis. 2026 Jul 8;17(1):851. doi: 10.1038/s41419-026-08883-y (PMC13631309; doi:10.1038/s41419-026-08883-y)
Supplement: Supplementary file 2 [file 41419_2026_8883_MOESM1_ESM.docx]

**Supplementary Materials.**

**Supplementary Figures.**

**Figure S1.** A. Quantification of the data shown in Figure 1L. B. Representative immunohistochemistry (IHC) staining images showing PRRC2A expression in TET and normal tissues (scale bar, 50 μm (left), 20 um (right)). C. Western blot analysis of PRRC2A protein levels in PRRC2A-knockdown and PRRC2A-overexpressing Ty-82, IU-TAB-1 and ThyL-6 cells. D. PRRC2A mRNA levels following PRRC2A knockdown in Ty-82, IU-TAB-1 and ThyL-6 cells. E. PRRC2A mRNA levels following PRRC2A overexpression in Ty-82, IU-TAB-1 and ThyL-6 cells. F-H. CCK-8 assays revealed the viability of Ty-82 (F), IU-TAB-1 (G), and ThyL-6 (H) cells following PRRC2A knockdown or overexpression. I-K. Colony formation assays revealed the proliferation of ThyL-6 cells following PRRC2A knockdown or overexpression. L-O. Transwell assays were employed to evaluate the migration and invasion ability of ThyL-6 cells with knockdown (L, M) or overexpression (N, O) of PRRC2A. Representative micrographs are presented (Scale bars: 50 μm).

**Figure S2.** A, B. Relative mRNA levels of four candidate genes, assessed by RT-qPCR in ThyL-6 cells following PRRC2A knockdown (A) or overexpression (B). C. Western blot analysis of NCOA4 protein levels in PRRC2A-knockdown and PRRC2A-overexpressing in ThyL-6 cells. D. Immunofluorescence analysis of NCOA4 protein green fluorescence in TET cells with PRRC2A knockdown or overexpression in IU-TAB-1cells (Scale bar: 10 μm). E. NCOA4 mRNA decay assay after actinomycin D treatment in IU-TAB-1 cells. F. Immunofluorescence analysis of NCOA4 protein green fluorescence in TET cells with PRRC2A knockdown or overexpression in ThyL-6 cells (Scale bar: 10 μm). G. NCOA4 mRNA decay assay after actinomycin D treatment in ThyL-6 cells. H. RIP-qPCR analysis showing PRRC2A enrichment on NCOA4 mRNA in ThyL-6 cells, with values presented relative to IgG control. I. MeRIP‐qPCR analysis was performed to examine the level of m6A in NCOA4 3’UTR in ThyL-6 cells with or without knockdown of PRRC2A or METTL3. J. Western blot analysis of METTL3, PRRC2A, and NCOA4 protein expression in ThyL-6 cells transfected with siMETTL3, shPRRC2A, or PRRC2A overexpression vector. ** P<0.01, ns, not significant.

**Figure S3.** A. Western blot analysis of NCOA4 protein levels in NCOA4-knockdown and NCOA4-overexpressing Ty-82, IU-TAB-1 and ThyL-6 cells. B, C. ECAR values of IU-TAB-1 cells with NCOA4 knockdown (B) or overexpression (C). D, E. ECAR values of ThyL-6 cells with NCOA4 knockdown (D) or overexpression (E). F. Western blotting analysis of NCOA4, LDHA, PKM2, GLUT1 and HK2 protein expression in ThyL-6 cells transfected with the knockdown or overexpression of NCOA4. G. FDP content, LDH activity, glucose uptake, lactate production, and acetyl-CoA levels in IU-TAB-1 and ThyL-6 cells with NCOA4 knockdown or overexpression. H. Immunofluorescence (IF) was used to validate the changes in LDHA protein levels (red fluorescent) in ThyL-6 cells with knockdown or overexpression of NCOA4. (Scale bar: 10 μm). I. EdU assays detecting the proliferation abilities of IU-TAB-1 cells following shNCOA4 and/or PRRC2A overexpression vector transfection. (Scale bar: 50 μm).

J. Transwell migration and invasion assays in IU-TAB-1 cells following shNCOA4 and/or PRRC2A overexpression vector transfection (Scale bar: 50 μm). K. EdU assays detecting the proliferation abilities of ThyL-6 cells following shNCOA4 and/or PRRC2A overexpression vector transfection. (Scale bar: 50 μm). L. Transwell migration and invasion assays in ThyL-6 cells following shNCOA4 and/or PRRC2A overexpression vector transfection (Scale bar: 50 μm). ** P<0.01.

**Figure S4.** A. The direct interaction between NCOA4 and PKM2 revealed by co-IP assays and analyzed by western blot assays in ThyL-6 cells. B. Representative immunofluorescence images showing the cellular localization of NCOA4 and PKM2 in ThyL-6 cells. (Scale bar: 3 μm). C. The ECAR values of the ThyL-6 cells with the knockdown of PKM2. D. The FDP content, LDH activity, glucose uptake, lactate fold, and level of acetyl-CoA of the ThyL-6 cells with the knockdown of PKM2. ** P<0.01.

**Figure S5.** A. Western blot analysis of PKM2 protein levels in ThyL-6 cells following knockdown. B. Measurement of extracellular acidification rate (ECAR) in ThyL-6 cells transfected with siPKM2 and/or NCOA4 overexpression vector. C. Western blot analysis of PKM2, STAT3, phosphorylated STAT3 (p-STAT3), c-Myc, LDHA, GLUT1, and HK2 protein levels in ThyL-6 cells transfected with siNC or siPKM2. D. Immunofluorescence (IF) analysis of LDHA protein expression (red fluorescence) in ThyL-6 cells transfected with siPKM2 and/or NCOA4 overexpression vector (Scale bar: 10 μm). E. EdU incorporation assays assessing the proliferative capacity of ThyL-6 cells with siPKM2 and/or NCOA4 overexpression vector (Scale bar: 50 μm). F-H. FDP content, LDH activity, glucose uptake, lactate production, and acetyl-CoA levels in Ty-82 (F), IU-TAB-1 (G) and ThyL-6 (H) cells with PKM2 knockdown and NCOA4 overexpression. I. Transwell migration and invasion assays in ThyL-6 cells following siPKM2 and/or NCOA4 overexpression vector transfection (Scale bar: 50 μm). J. Quantification of EdU-positive rates shown in Figures 7J, K and Figure S5E. K. Quantification of migration TET cells shown in Figures 7L, M and Figure S5I.

L. Quantification of invasion TET cells shown in Figures 7 M and Figure S5I. ** P<0.01, ns, not significant.

**Supplementary Tables.**

**Table S1. PRRC2A expression in TET and clinicopathological characteristics (n=54)**

|  | PRRC2A expression | |  |
| --- | --- | --- | --- |
|  | Low (n=35) | High (n=19) | p value |
| Gender |  |  | 0.80 |
| Male | 16 | 8 |  |
| Female | 19 | 11 |  |
| Age(years) |  |  | 0.82 |
| ＜ 60 | 25 | 13 |  |
| ≥ 60 | 10 | 6 |  |
| Histological subtype |  |  | 0.428 |
| Type A thymoma | 8 | 4 |  |
| Type B1 thymoma | 4 | 2 |  |
| Type B2 thymoma | 3 | 3 |  |
| Type B3 thymoma | 1 | 1 |  |
| Type AB thymoma | 17 | 5 |  |
| Thymic carcinoma | 2 | 4 |  |
| T stage |  |  | 0.002 |
| T1 | 26 | 7 |  |
| T2 | 7 | 2 |  |
| T3 | 2 | 8 |  |
| T4 | 0 | 2 |  |
| M stage |  |  | 0.004 |
| M0 | 35 | 17 |  |
| M1 | 0 | 2 |  |
| TNM stage |  |  | 0.006 |
| I | 23 | 7 |  |
| II | 10 | 2 |  |
| III | 2 | 8 |  |
| IV | 0 | 2 |  |
| Masaoka–Koga stage |  |  | 0.008 |
| I | 20 | 5 |  |
| II | 13 | 6 |  |
| III | 2 | 6 |  |
| IV | 0 | 2 |  |
| Myasthenia gravis |  |  | 0.76 |
| Yes | 12 | 5 |  |
| No | 23 | 14 |  |

**Table S2. The sequences of shRNAs or siRNAs.**

| Name | Sequences |
| --- | --- |
| shPRRC2A-1 | 5′-GAGGATGAGAGTGTTAGATTA-3′ |
| shPRRC2A-2 | 5′-GCAGATTCTCAAGTTGAAGAT-3′ |
| shPRRC2A-3 | 5′-GCTGGTGCTGAAGGATTATTA-3′ |
| shNCOA4-1 | 5′-GCTGCTCTATGAACCTCTTAA-3′ |
| shNCOA4-2 | 5′-GGAAGTGATTTCAGTGTTGAA-3′ |
| shNCOA4-3 | 5′-CCAGCAGATCTGCTTAGAGAA-3′ |
| siMETTLE3-1 | 5′-GGAAGAGCUGCUUUACAAATT-3′ |
| siMETTLE3-2 | 5′-GCGCUUACUGCUUAGUUAUTT-3′ |
| siMETTLE3-3 | 5′-CCACCUCACAAACUAACAUTT-3′ |
| siPKM2-1 | 5′-GCGAGGAAAGTGAAGCGAATT-3′ |
| siPKM2-2 | 5′-GCACGAAGCAGACATTGACTT-3′ |
| siPKM2-3 | 5′-GGUUGAGAAUUGUAGUACATT-3′ |

**Table S3. The antibodies used for western blot, immunofluorescence staining, and immunohistochemistry.**

| Antibody | Company | Cat.No. |
| --- | --- | --- |
| PRRC2A | Santa Cruz | sc-373747 |
| NCOA4 | Abcam | ab314553 |
| METTL3 | Proteintech | 15073-1-AP |
| LDHA | Proteintech | 21799-1-AP |
| GLUT1 | Proteintech | 66290-1-Ig |
| PKM2 | Proteintech | 15822-1-AP |
| STAT3 | Cell Signaling | 9139 |
| p-STAT3 | Cell Signaling | 9131 |
| c-Myc | Cell Signaling | 5605 |
| Ki-67 | Proteintech | 27309-1-AP |
| β-actin | Proteintech | 66009-1-Ig |
| m6A | Synaptic Systems | 202003 |
| Flag | Proteintech | 20543-1-AP |
| His | Proteintech | 66005-1-Ig |

**Table S4. Primers for qRT-PCR.**

|  | Primer name | Sequence (5’-3’) |
| --- | --- | --- |
|  | PRRC2A-F | AGGGCAAGTCCTTAGAGATCC |
|  | PRRC2A-R | TTCAGGCTTGGAAGGTTGGC |
|  | GABRD-F | AGTGACAGGGCTGTTTCTGG |
|  | GABRD-R | TCTTCTCGGTAGGTGGTGGA |
|  | NCOA4-F | TGCAGAGAGGAACTTGAGGA |
|  | NCOA4-R | GTCTTCTGGCAGTGTAGGTG |
|  | RER1-F | CCTGCTGATGCTGGTGAACT |
|  | RER1-R | AGGTAGGCGTTGTAGAGGGA |
|  | NIPAL3-F | TCTTCCTGGTGACCTTCGTC |
|  | NIPAL3-R | AGGATGACAGGCACACTTGA |
|  | METTL3-F | AGGAGCTGTGCAGTAGGCAA |
|  | METTL3-R | TCCCGCTTCCAAATGTCACT |
|  | PKM2-F | TTTGAGGAACTCCGCCACAG |
|  | PKM2-R | GACACAGTCCATGCCATTCAC |

**Table S5: NCOA4-IP enrichment proteins (Top 100)**

| Gene Symbol | Abundance | Score |
| --- | --- | --- |
| PKM2 | 7409671090 | 433.82 |
| PLEC | 7262232215 | 394.59 |
| ACTB | 4.195E+10 | 332.41 |
| TUBB | 6382279282 | 179.55 |
| HSPA8 | 2362454600 | 143.13 |
| TUBB4B | 202929940 | 135.98 |
| ACTA1 | 444765705 | 121.18 |
| CLTC | 1870235334 | 117.98 |
| TUBA1B | 4805896068 | 117.09 |
| TUBB4A | 23274114 | 110.79 |
| HSPB1 | 1.4753E+10 | 82.17 |
| TUBB8 | 45164214 | 78.09 |
| HSPA1B | 1481666412 | 74.43 |
| POTEE | 31756520 | 70.62 |
| EPPK1 | 589119047 | 70.11 |
| NME2 | 1662031078 | 67.01 |
| ATP5F1B | 1191282180 | 66.8 |
| GAPDH | 2133565901 | 66.25 |
| NME1 | 107369040 | 65.22 |
| ACTBL2 | 47214800 | 62.45 |
| HSPD1 | 1366242100 | 62.16 |
| ACTN4 | 568037078 | 51.36 |
| RPS10 | 1223738806 | 48.97 |
| ATP5F1A | 707836799 | 48.75 |
| CALM3 | 1550790816 | 45.19 |
| FASN | 471521625 | 44.62 |
| MDH2 | 728619654 | 43.8 |
| FAM83H | 492131939 | 42.68 |
| ACTN1 | 12142115 | 41.09 |
| HBA1; HBA2 | 795869927 | 38.72 |
| HSP90AA1 | 427864797 | 38.48 |
| EEF1A1 | 804105726 | 38.35 |
| HSP90AB1 | 1092850253 | 37.69 |
| DNPEP | 757996126 | 35.09 |
| PHB1 | 393347198 | 35.04 |
| ALB | 1705733388 | 34.55 |
| ALDOA | 672661177 | 33.98 |
| RPSA | 687502664 | 33.22 |
| HSPA5 | 242610386 | 32.43 |
| HBB | 1196484159 | 32.33 |
| PHB2 | 537780845 | 30.74 |
| EEF1G | 590331408 | 30.38 |
| HSPA9 | 746718159 | 29.99 |
| PSMD2 | 192483706 | 29.06 |
| EEF1A2 | 2366737763 | 28.93 |
| RPS16 | 602185404 | 28.35 |
| ST14 | 297445052 | 27.52 |
| CCT8 | 384133137 | 27.09 |
| RANGAP1 | 506041215 | 25.61 |
| RPS19 | 1352670754 | 25.16 |
| SLC25A5 | 607702628 | 24.2 |
| S100A9 | 253060040 | 23.79 |
| PRDX2 | 377312876 | 23.22 |
| PABPC1 | 252209719 | 22.69 |
| KPNB1 | 362603659 | 22.68 |
| SLC25A6 | 140786584 | 22.53 |
| AHCY | 167705946 | 22.3 |
| CAND1 | 95372579 | 21.8 |
| RPS3 | 779717852 | 21.39 |
| CBR1 | 302825656 | 21.15 |
| JUP | 236128863 | 20.92 |
| ARPC4 | 306066132 | 20.85 |
| CSNK1A1 | 326022086 | 20.51 |
| PRDX1 | 592139352 | 19.08 |
| ENO1 | 303877595 | 18.99 |
| UQCRC2 | 79021670 | 18.42 |
| TRIM28 | 253619154 | 18.27 |
| CCT4 | 189402573 | 17.65 |
| PDIA6 | 198605508 | 17.25 |
| CCT2 | 166059072 | 17.06 |
| HADHA | 263559290 | 17 |
| HADHB | 356491744 | 16.59 |
| RPL12 | 2020691593 | 16.46 |
| S100A7 | 316971882 | 16.2 |
| GOT2 | 231118458 | 16.18 |
| GPI | 141411875 | 16.15 |
| LDHA | 465040151 | 16 |
| TFRC | 192252435 | 15.59 |
| OGT | 94368899.5 | 15.33 |
| NONO | 71598872 | 14.87 |
| RPLP2 | 896063648 | 14.8 |
| IMMT | 117144385 | 14.43 |
| SLC3A2 | 259848132 | 14.34 |
| NUDT21 | 186442846 | 13.89 |
| RPS20 | 482164450 | 13.65 |
| SHMT2 | 216409436 | 13.36 |
| UGDH | 106350154 | 13.2 |
| RPL29 | 52037508 | 12.98 |
| CAPZA2 | 117181652 | 12.83 |
| TKT | 149758088 | 12.56 |
| ANXA2 | 115311640 | 12.55 |
| PHGDH | 157977676 | 12.5 |
| H4C1 | 201298380 | 12.31 |
| RPLP0 | 796154320 | 12.29 |
| RPS25 | 958910591 | 12.19 |
| RPLP1 | 486167870 | 12.1 |
| MGST1 | 45239206 | 11.66 |
| EEF2 | 153848539 | 11.51 |
| SNRPB | 201215736 | 11.47 |
